# Supersedure, mites, and visible disease in *Apis mellifera* (Hymenoptera: Apidae) colonies explain differences in productivity and survival, but the effects may be difficult to see

**DOI:** 10.1093/jee/toaf094

**Published:** 2025-06-13

**Authors:** Michael Peirson, Abdullah Ibrahim, Lynae P Ovinge, Shelley E Hoover, Stephen F Pernal

**Affiliations:** Agriculture and Agri-Food Canada, Beaverlodge Research Farm, Beaverlodge, Alberta, Canada; Agriculture and Agri-Food Canada, Beaverlodge Research Farm, Beaverlodge, Alberta, Canada; Alberta Agriculture and Forestry, Lethbridge Research Centre, Lethbridge, Alberta, Canada; Department of Biological Sciences, University of Lethbridge, Lethbridge, Alberta, Canada; Alberta Agriculture and Forestry, Lethbridge Research Centre, Lethbridge, Alberta, Canada; Department of Biological Sciences, University of Lethbridge, Lethbridge, Alberta, Canada; Agriculture and Agri-Food Canada, Beaverlodge Research Farm, Beaverlodge, Alberta, Canada

**Keywords:** varroa, supersedure, chalkbrood, fumagillin, protein

## Abstract

We investigated whether field assessments of honey bee (*Apis mellifera* L.) colony health explain subsequent colony size, honey production, and survival. Field detections of visible diseases, *Varroa destructor* (Anderson and Trueman) and queen replacement events were recorded during a multisite cohort study, which also incorporated fumagillin and protein supplementation as colony-level treatments. Together, treatment groups and field observations explained between 5% of the variability in adult bee counts and 28% of the variability in honey production among colonies, after accounting for the effects of region and date. In particular, detections of minor disease symptoms, mainly chalkbrood, were associated with large reductions in honey production and approximately doubled the short-term probability of colony death. Although the effects of treatments and field-observed events were significant, unexplained variability among similarly managed colonies was much greater. Consequently, beekeepers may be unable to detect the effects of these field-observable factors, or distinguish effective treatments from ineffective ones. Despite this, interventions to reduce the prevalence of varroa and visible diseases, and to prevent queen loss, are likely to improve honey bee health and productivity.

## Introduction

In recent years, high death rates among honey bee (*Apis mellifera*) colonies have been regularly reported (Pirk et al. 2014, Bruckner et al. 2023, CAPA 2023, Gray et al. 2023, Requier et al. 2024). This mortality is attributed to multiple stressors acting in concert. Suggested causes include parasites, diseases, pesticides, poor nutrition, management, queen quality, genetics, and weather conditions (Steinhauer et al. 2018, Hristov et al. 2020, French et al. 2024).

To investigate the problem, beekeepers have often been asked to report their colony losses and opinions about those losses (Pirk et al. 2014, Bruckner et al. 2023, CAPA 2023, Gray et al. 2023, Requier et al. 2024). This type of study tends to produce results that are difficult to interpret. For example, beekeepers regularly report “poor queens” (CAPA 2023), even though they cannot directly observe the quality of a queen, and defects that can be observed, such as queen death or poor colony growth rate, often have causes unrelated to queen quality. Alternatively, some studies have compared the pathogen loads and pesticide exposures of apparently successful and unsuccessful colonies (vanEngelsdorp et al. 2009, Cornman et al. 2012) or have tried to relate observations of colony health to longer term outcomes (Genersch et al. 2010, Guzmán-Novoa et al. 2010, vanEngelsdorp et al. 2013, van Dooremalen and van Langevelde, 2021). Surprisingly, studies do not usually quantify the relative importance of contributing factors, and in many cases colony death is the only outcome considered.

In our earlier article (Peirson et al. 2024), we showed that 2 common beekeeper-provided treatments, protein supplements and fumagillin, produced effects that were inconsistent among regions and dates and small compared to the overall variability among colonies. Here we use additional data from the same large-scale experiment to investigate whether events that can be observed by a beekeeper—the detection of *Varroa destructor*, the detection of visible diseases, and queen supersedure—were associated with colony size and were useful predictors of future colony growth, honey production, and survival.

## Methods

A comprehensive description of the study can be found in Peirson et al. (2024). Briefly, 362 honey bee colonies were situated in 3 regions: Southern Alberta (SAB), Northern Alberta (NAB), and Prince Edward Island (PEI), with one beekeeping operation per region. Three hundred and sixteen of the colonies were present from the outset of the experiment and inspected repeatedly for evidence of queen replacement, disease, and *Varroa destructor*. Using a factorial design, colonies were either fed or deprived of a protein supplement containing 25% pollen (Global Patties, Airdrie, AB) during most of the beekeeping season outside of peak bloom, and were either treated or not treated with label-dose applications of fumagillin (Fumagilin-B; Medivet Pharmaceuticals, High River, AB) in the fall of each year. In other respects, colonies were managed according to the standard practices of the local commercial beekeeper.

Adult bee and sealed brood measurements were obtained at inspection dates in May, June, and August beginning in May 2014 and ending in May 2016, except that inspections of the PEI colonies began in June 2014 (Supplementary Fig. 1). Adult bee measurements were obtained between dawn and the beginning of flight activity and sealed brood measurements of the same colonies were obtained later the same day. In 2014, the area covered by adult bees or sealed brood was visually estimated by trained observers using 1 inch (2.54 cm) square grids overlaid on the frames, and was subsequently converted to bee and brood counts. In 2015 and 2016, photographs were taken of the frames and the bees or brood were counted with the aid of software (Honeybee Complete 4.2, WSC Scientific GmbH, Heidleberg Germany). For additional methodological details, please refer to Peirson et al. (2024).

### Queen and Disease Inspections

Following measurements of colony size, and also during inspections before and after winter, frames of the brood nest were examined to locate the queen and to identify symptoms of diseases in adult bees. Paint-marked and wing-clipped queens were used to ensure that queen loss would be detected. If an unmarked queen was found, the new queen was captured and marked. Natural and beekeeper-initiated queen replacement events were recorded. We did not attempt to distinguish among types of natural queen replacement events (emergency queen replacement, supersedure, swarms) and refer to them collectively as supersedure events. Beekeeper-initiated queen replacements were a small proportion of the total (13 of 181 cases). Once the queen had been found, a sample of adult bees was taken from a frame that contained brood and placed immediately on dry ice, then stored at −80 °C when returned to the laboratory. Approximately 200 bees from this sample were washed in 70% ethanol to measure *V. destructor* infestation levels (de Jong et al. 1982). Next, adult bees were shaken or brushed off and all brood frames were examined for symptoms of disease, with the following diseases recorded: American Foulbrood (AFB), European Foulbrood (EFB), Chalkbrood, Sacbrood, Deformed Winged Virus, or Other. “Other” was a general category to capture additional symptoms or pests observed during an inspection; examples included hygienic removal of apparently healthy brood, paralyzed bees, and mites directly observed by the inspector. For each disease, inspection results were classed in 1 of 4 categories: none, low (1 to 10 symptomatic individuals), medium (11 to 100), or high (> 100).

### Statistical Analysis

All statistical analyses were conducted using R (version 4.4.1; R Core Team 2024) and R Studio (version 2024.09.0 + 375; Posit Team 2024). For models, the nlme package (version 3.1-166; Pinheiro et al. 2024) and the survival package (version 3.7-0; Therneau 2024) were used. Estimates and contrasts from the models were obtained using the emmeans package (Lenth 2024). Estimates of *R*^2^ for mixed models were obtained using the MuMIn package (version 1.48.4; Bartoń 2024). The output of the statistical analyses can be found in Supplementary file S1; the analysis code (readable by R Studio) in Supplementary file S2; and the dataset in Supplementary file S3.

Each of the field-observed variables (supersedure, varroa detection, disease detection) was modeled as binary (detected or not-detected at a given date) even though we had quantitative data for varroa and semiquantitative data for disease. Because all the higher mite-count samples occurred in one region (PEI), analysis of varroa as a continuous variable would have produced estimates controlled by outcomes in that region only. Analysis of disease or varroa as ordinal variables was impossible because there were few or no cases of some levels of these variables at some combinations of region, date, and treatment group.

Variance explained by the statistical models was estimated using Nakagawa and Schielzeth’s modification of the *R*^2^ statistic for use with mixed models (Nakagawa and Schielzeth 2013, Nakagawa et al. 2017). For each dependent variable, a reference maximum likelihood model with one random effect, colony, and 3 fixed effects: region, date, and the interaction of region and date was developed, and the proportion of variance explained by the fixed effects, *R*^2^_(m)_, was calculated. In this model, *R*^2^_(m)_ is the variance explained by region and date and 1 − *R*^2^_(m)_ is the proportion of variance not explained by region and date; in other words, the variance among colonies observed at the same region and date. Each predictor (detection of supersedure, disease, or varroa) and its interactions with region and date was added to the model and *R*^2^_(m)_ calculated again. Each factor is reported as the percent reduction in the unexplained variance following the addition of the factor to the model; that is, as the factor’s apparent contribution to variance among colonies at the same region and date.

We also estimated the contributions of the protein and fumagillin treatment groups to variance within region and date. These estimates used maximum likelihood versions of the mixed models developed in our previous paper (Peirson et al. 2024). In addition, an “all-factors” model was developed for each dependent variable. The all-factors model included all interactions of the design factors: region, date, protein treatment, and fumagillin treatment, as well as all 2-way interactions between varroa detection, disease detection, or queen-event detection with the design factors. Interactions among field-observed factors and interactions between a field-observed factor and more than one design factor were excluded because in most cases such models produced singularities. Colony was considered a random factor. Autoregressive (AR(1)) correlations were included and variances were estimated separately for each region and date combination. The models were reduced by successively removing nonsignificant higher-order interactions until only significant effects and main effects remained, except that interactions between fumagillin and date were always retained to reflect the fact that the initial fumagillin treatment occurred after the first summer.

The Cox proportional hazards models for colony survival were developed from the corresponding models in Peirson et al. (2024), modified to allow the inclusion of field-observable events as time-dependent covariates. Before removal of nonsignificant interactions, the model included all interactions of the design factors (region, protein, and fumagillin treatments), and the interaction of the field-observable events with the design factors. Region was a stratified factor and fumagillin treatment was a time-dependent covariate, to account for its date of application. Varroa and disease have been treated as influencing the risk of death between the date of detection and the subsequent colony health inspection. For example, if a disease was detected only at the June 2014 inspection, the colony was modeled as “Sick” for the period between June and August; subsequently it was modeled as “Healthy”. Queen events were also treated as influencing the risk of death for one inspection period, but the influence of a queen event was not taken to begin until the inspection following its detection. This was necessary because when a queen loss led directly to colony death, the date of the queen event was considered the date of death and consequently there were no cases in the dataset where a queen event on one date was followed by a colony death on the next date. As such, the model tests the effect of a recent successful queen replacement on the risk of colony death.

## Results

### Varroa destructor

A total of 2,120 samples, more than 450,000 bees in total, were washed in 70% ethanol, and 1,247 varroa mites detected. Most of the mites (1,074) were found at the PEI beekeeping operation. The average sample there contained 1.7 ± 4.0 mites per hundred bees (mean ± SD), far more than at NAB (0.08 ± 0.25) or SAB (0.02 ± 0.17)). Each operation differed significantly from the others (NAB vs SAB: *t* = −5.2, df = 1426, *P* < 0.001; SAB vs PEI: *t* = −8.3, df = 366, *P* < 0.001; PEI vs NAB: 8.0, df = 366, *P* < 0.001). In addition, there were many significant differences among dates, which largely reflected the timing of miticide applications. There was no difference in mite levels between the fumagillin-treated and nontreated groups (*t* = 1.03, df = 2063, *P* = 0.30). Protein-supplemented colonies contained more mites than nonsupplemented colonies (*t* = 2.00, df = 1589, *P* = 0.045). However, mite counts were not normally distributed either among regions and dates, or among colonies within a region and date (Supplementary file S1 - Fig. S1.18). The elevated mite levels occurred in a few protein-supplemented colonies in PEI during the first year of the study (Supplementary file S1 - Fig. S1.19). As this occurred at the beginning of the study and was not apparent later, the protein treatment cannot have caused the high mite loads, and since the high mite loads occurred in only a few colonies out of 316, they are unlikely to have affected estimates of the effect of protein.

### Visible Diseases

Though disease occurred in all regions, symptoms were not commonly found. Of the 2,112 field inspections conducted on viable colonies, 1,842 (87%) found no diseases, 241 (11%) found 1 disease, and 29 (1%) detected 2 or more diseases. However, 69% (83 of 121) of colonies in PEI, 57% (67 of 117) of colonies in SAB, and 13% (16 of 123) colonies in NAB were visibly diseased at least once (Table 1). There were differences among regions in the number of colonies that were ever visibly diseased (*χ*^2^ = 85, df = 2, *P* < 0.001), and in the proportion of inspections at which disease was found (*χ*^2^ = 307, df = 2, *P* < 0.001), with the most frequent detections occurring at PEI (131 of 322 inspections) and the least frequent at NAB (20 of 842 inspections). The frequency of disease detection also differed among dates (*χ*^2^ = 111, df = 8, *P* < 0.001). Of the 270 inspections where disease was found, 106 were in June and only 5 were in November. Neither treatment (supplemental feeding nor Fumagilin-B application) influenced the number of visibly diseased colonies.

**Table 1.** Number of diseases per colony and per inspection

	Number of diseases found							
Subgroup	During the whole experiment				During a single inspection			
	0	1	2	3	0	*1*	2	3
	Number (Percent) of colonies				Number (Percent) of inspections			
*Region*								
SAB	50 (43)	59 (50)	8 (7)	0 (0)	829 (87)	116 (12)	3 (0)	0 (0)
NAB	107 (87)	15 (12)	1 (1)	0 (0)	822 (98)	20 (2)	0 (0)	0 (0)
PEI	38 (31)	43 (35)	31 (26)	9 (7)	191 (59)	105 (33)	23 (7)	3 (1)

Treatments								
None	50 (57)	26 (30)	10 (11)	2 (2)	478 (89)	53 (10)	6 (1)	0 (0)
Fumagillin	42 (47)	31 (35)	15 (17)	1 (1)	501 (87)	68 (12)	7 (1)	1 (0)
Protein	43 (48)	36 (40)	7 (8)	3 (3)	391 (84)	68 (15)	5 (1)	2 (0)
Both	60 (63)	24 (25)	8 (8)	3 (3)	472 (89)	52 (10)	8 (2)	0 (0)

**Fig. 1.**
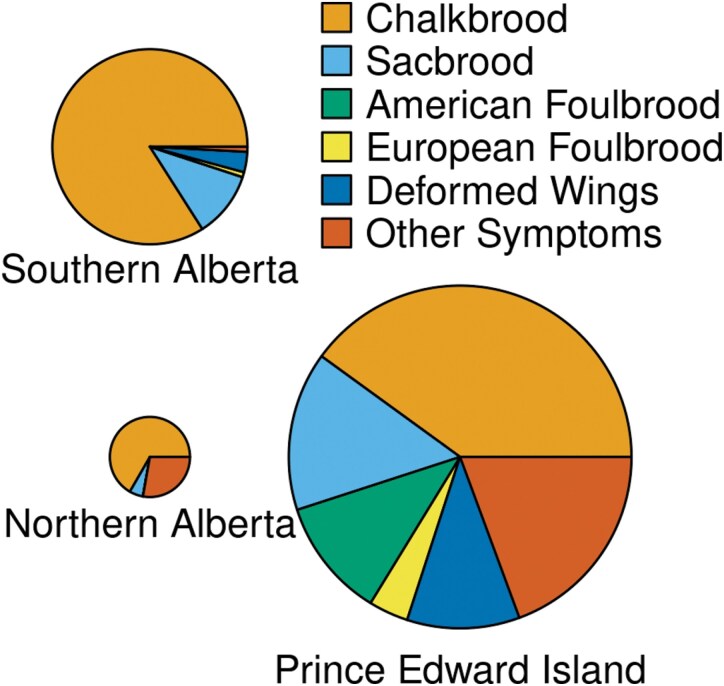
There were large differences in the prevalence and types of disease among regions. The areas of the circles are proportionate to the number of disease detections per colony inspection. Only data from the May, June, and August assessments are shown because November and April inspections in PEI did not include a disease assessment. Results shown summarize 119 disease detections during 739 colony inspections (SAB), 18 detections from 663 inspections (NAB), and 160 detections from 322 inspections (PEI).

**Fig. 2.**
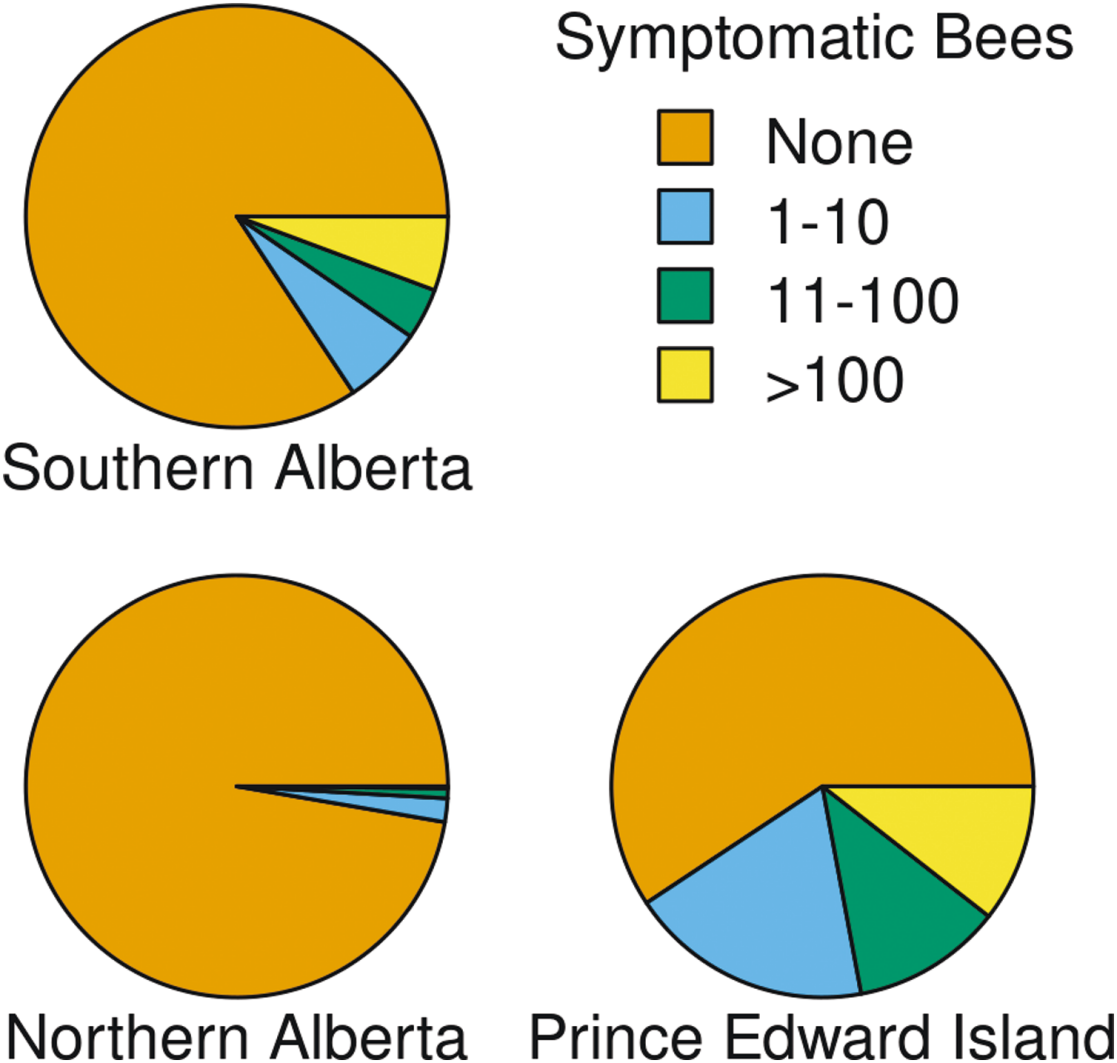
Intensity categories of symptomatic bees per colony inspection, by region. When more than one disease was detected at a single inspection, only the disease with the larger number of symptomatic individuals is shown. Only data from the May, June, and August assessments are shown because November and April inspections in PEI did not include a disease assessment. Results shown summarize 739 colony inspections (SAB), 663 inspections (NAB) and 322 inspections (PEI).

In Alberta, most diseased colonies (SAB: 59/67; NAB: 15/16) had symptoms of only 1 disease over the duration of the study, but nearly half (40 of 83) of affected colonies in PEI had 2 or more observable diseases (Table 1). Chalkbrood was most frequently observed (179 inspections), followed by sacbrood (38), other (37), bees with deformed wings (23), AFB (18), and EFB (7). Chalkbrood accounted for 87% (SAB) and 60% (NAB) of disease detections in Alberta, but only 49% of detections in PEI. All the AFB and all but 1 of the EFB detections were at the PEI location (Fig. 1). Most colonies were symptomatic for only 1 or 2 inspections and appeared healthy afterward. When disease was apparent, it appeared mild or moderate in most cases, with 153 observations classified as low, 82 as medium, and only 84 as high (Fig. 2). Protein and fumagillin treatments did not affect visible disease symptoms (protein: *χ*^2^ = 0.81, df = 1, *P* = 0.37; fumagillin: *χ*^2^ = 0.47, df = 1, *P* = 0.49).

### Association between Field-observable Events or Treatments and Colony Size

Most of the variability in adult bee counts, 79% (Table 2), could be explained by the fixed effects of the reference model, that is by region, date, and the interaction of region and date. About half of the variability in sealed brood counts, 49% (Table 3), could be explained by the same factors.

**Table 2.** Comparison of maximum likelihood models of adult bee count

Fixed effects	AIC^a^	Likelihood ratio	*P*	Coefficient of determination *R*^2^_(m)_^b^	Percent reduction in unexplained variability (fixed effects)^c^	Coefficient of determination *R*^2^_(c)_^b^	Percent reduction in unexplained variability (total)^c^
Region & Date	27,673			0.7868		0.8250	
Region & Date + Varroa	27,663	26.19	0.001	0.7889	1.0	0.8256	0.3
Region & Date + Disease	27,675	14.72	0.065	0.7952	3.9	0.8354	5.9
Region & Date + Queen Event	27,671	18.61	0.017	0.7895	1.2	0.8231	-1.1
Region & Date + Treatments	27,689	71.94	0.057	0.7913	2.1	0.8313	3.6
All factors	27,656	65.38	<0.001	0.7977	5.1	0.8351	5.7

Models included 2-factor interactions of fixed effects, colony as a random effect, and autoregressive correlations. The model with treatments is the optimized model presented in our previous publication (Peirson et al. 2024), but for comparison with the other models has been run as a maximum likelihood model using the reduced dataset which arises from excluding missing observations of varroa and disease. The all-factors model is the optimized model described in the text except that here it is a maximum likelihood model, to permit comparisons against the other models.

^a^Akaike Information Criterion.

^b^See Nakagawa and Schielzeth (2013). *R*^2^_(m)_ gives the proportion of the variance which is explained by the fixed effects of a mixed model. *R*^2^_(c)_ gives the proportion of the variance which is explained by the total model. 1 - *R*^2^_(m)_ gives the proportion of variance not explained by the fixed effects of the model, which in the case of the region and date model is the proportion of variance due to differences among colonies at the same region and date.

^c^Equals (100 * (*R*^2^(indicated model) − *R*^2^(region & date model))/(1 − *R*^2^(region & date model)).

**Table 3.** Comparison of maximum likelihood models of sealed brood count

Fixed effects	AIC^a^	Likelihood ratio	*P*	Coefficient of determination *R*^2^_(m)_^b^	Percent reduction in unexplained variability (fixed effects)^c^	Coefficient of Determination *R*^2^_(c)_^b^	Percent reduction in unexplained variability (total)^c^
Region & Date	26,527			0.4854		0.5533	
Region & Date + Varroa	26,521	21.85	0.005	0.4897	0.8	0.5621	2.0
Region & Date + Disease	26,532	10.82	0.212	0.4972	2.3	0.5677	3.2
Region & Date + Queen Event	26,413	130.2	<0.001	0.5232	7.4	0.5696	3.7
Region & Date + Treatments	26,530	53.26	0.003	0.5418	11.0	0.6109	12.9
All factors	26,399	190.7	<0.001	0.5849	19.3	0.6411	19.7

Models included 2-factor interactions of fixed effects, colony as a random effect, and autoregressive correlations. The model for treatment is the optimized model presented in Peirson et al. (2024), but for comparison with the other models is run as a maximum likelihood model using the reduced dataset which arises from excluding missing observations of varroa and disease. The all-factors model is the optimized model described in the text except that here it is a maximum likelihood model, to permit comparisons against the other models.

^a^Akaike Information Criterion.

^b^See Nakagawa and Schielzeth (2013). *R*^2^_(m)_ gives the proportion of the variance which is explained by the fixed effects of a mixed model. *R*^2^(c) gives the proportion of the variance which is explained by the total model.

^c^Equals (100 * (*R*^2^(indicated model) − *R*^2^(region & date model))/(1 − *R*^2^(region & date model)).

Models which accounted for field-observable events were significantly (*P* < 0.05) or near significantly (*P* < 0.1) better than the reference model in most cases (Tables 2 and 3). Similarly, the model that included treatment effects (supplemental protein and fumagillin) was significantly better than the reference model (region, date, and region * date) for sealed brood (*P* = 0.003) and marginally significantly better for adult bees (*P* = 0.057). The model that included all factors represented the best overall fit (lowest Akaike Information Criterion (AIC) values; *P* < 0.001 for both adults and sealed brood; Tables 2 and 3). These observations suggest that there were real relationships between the field-observed factors and both measures of colony population.

Despite being significant, the field-observable events and treatments only modestly increased the estimates of *R*^2^_(m)_. For adult bees, the fixed effects of the reference model (region, date, and the interaction of region and date) accounted for 78.7% of the total variability, while the all-factors model explained 79.8%; this represents only a 5% reduction in unexplained variability. For sealed brood, the all-factors model reduced the amount of unexplained variability by about 19%.

The effects of protein and fumagillin were described in our previous report (Peirson et al. 2024). In addition, each type of field-observable event (detection of visible disease, detection of varroa, queen supersedure) had a significant relationship to colony size. For varroa, this relationship appeared as interactions with region and with date (adult bees: varroa * date: *F* = 3.53, df = 5, 1035, *P* = 0.004; adult bees: varroa * region: *F* = 3.60, df = 2, 1035, *P* = 0.028; sealed brood: varroa * region: *F* = 3.41, df = 2, 1004, *P* = 0.034). In June 2014, colonies in which varroa was detected had more adult bees (difference averaged across region: 2,151 ± 810 bees; *t* = 2.654, df = 1035, *P* = 0.008). In PEI, colonies in which varroa was detected were significantly smaller than other colonies on the date of the detection (averaged across all dates of detection, difference in adult bees: −1,768 ± 699 bees; *t* = *−*2.53, df = 1035, *P* = 0.011; difference in sealed brood cells: −1,910 ± 590; *t* = *−*3.3, df = 1023, *P* = 0.001)).

**Fig. 3.**
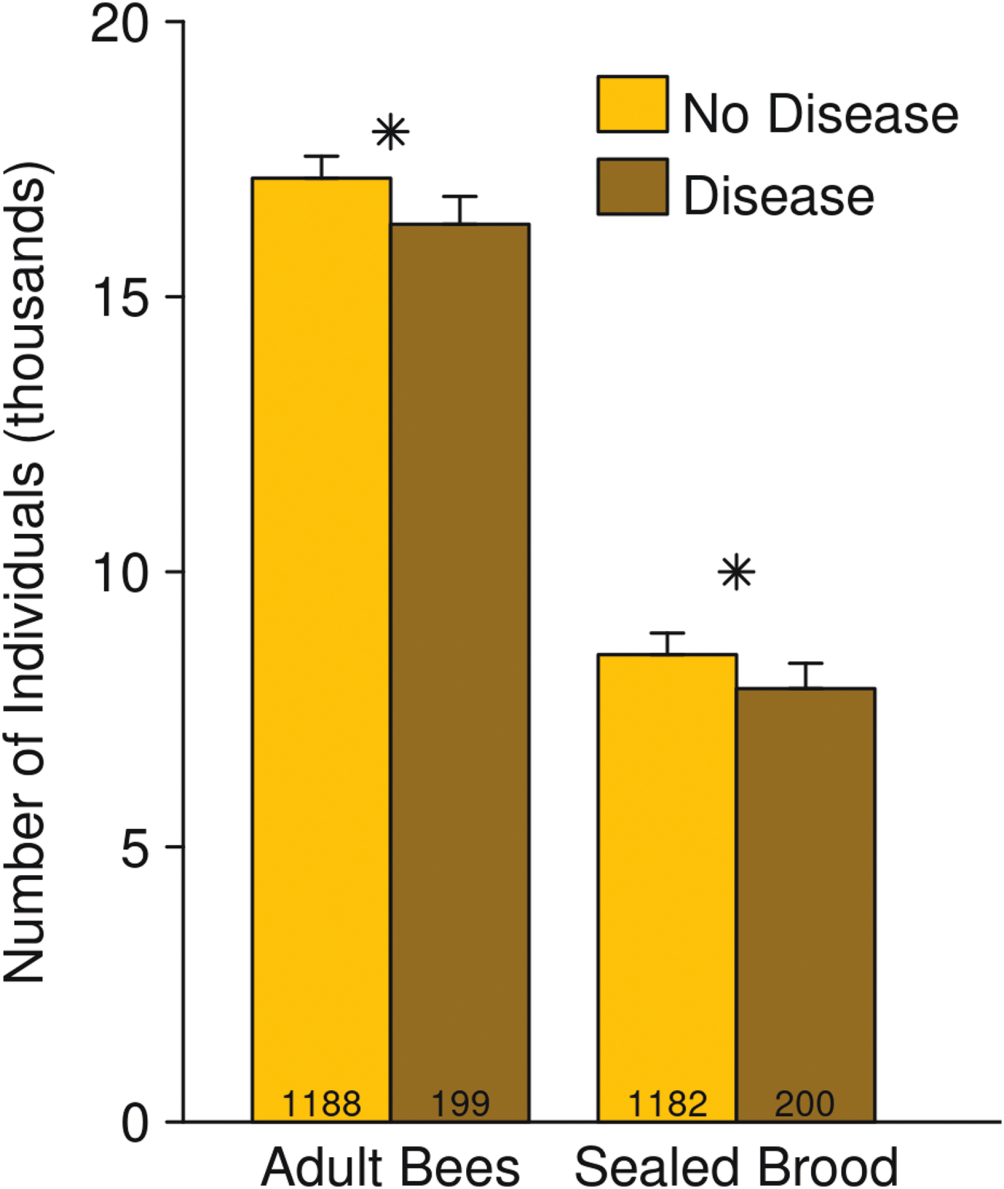
“Sick” colonies were smaller than “healthy” colonies on the date of the disease detection, having fewer adult bees and sealed brood cells. Emmeans ± SE are shown, as estimated from the “all-factors” models for adult bees and sealed brood. Stars indicate a statistically significant difference (*P* < 0.05) between the emmeans of sick and healthy colonies. The number of colony inspections used for estimates of diseased and nondiseased colonies are indicated at the bottoms of the bars.

Colonies with visible disease were modestly smaller than healthy colonies on the date of the disease detection (Fig. 3; difference in adult bees: −836 ± 348, *F* = 5.77, df = 1, 1035, *P* = 0.016; *t* = −2.403, df = 1035, *P* = 0.016; difference in sealed brood cells: −620 ± 280; *F* = 4.9, df = 1, 1023, *P* = 0.027; *t* = −2.2, df = 1023, *P* = 0.027)). Colonies which had experienced a recent queen event also tended to be smaller on the date the queen event was detected (ie immediately after the queen event), than those which had not (difference in adult bees: −1,670 ± 580, *F* = 8.314, df = 1, 1035, *P* = 0.004; *t* = 2.88, df = 1035, *P* = 0.004; difference in sealed brood cells: −2800 ± 760; *F* = 6.4, df = 1, 1023, *P* = 0.012; *t* = −3.7, df = 1023, *P* < 0.001). Queen events reduced the number of sealed brood cells but the amount of the reduction varied among regions and dates (queen event * date: *F* = 4.3, df = 5, 1023, *P* < 0.001; queen event * region: *F* = 13, df = 5, 1023, *P* < 0.001). When averaged across sampling dates, the reduction in sealed brood cells after a queen event was 5,200 ± 800 in NAB but only1,300 ± 900 in SAB; when averaged across regions, a reduction of 4,800 ± 700 cells was seen in August 2014, but only 940 ± 700 cells in August 2015.

### Relative Colony Size

To distinguish the effects of events and treatments from the effects of preexisting colony size, sealed brood counts were expressed as a percentage of adult bee counts on the same date and adult bee counts were expressed as a percentage of the adult bee count on the prior inspection date. When brood production was expressed in this way, region, date, and the interaction of region and date, accounted for 64% of the variability in the dataset (Table 4), while the all-factors model accounted for 67% of the variability and was a significantly better fit. Similarly, the model for adults that included all factors was a better fit than the model based on region and date alone (Table 4).

**Table 4.** Comparison of maximum likelihood models of relative colony population

Fixed effects	AIC^a^	Likelihood ratio	*P*	Coefficient of determination *R*^2^_(m)_^b^	Percent reduction in unexplained variability (fixed effects)^c^	Coefficient of determination *R*^2^_(c)_^b^	Percent reduction in unexplained variability (total)^c^
*Relative sealed brood models*							
Region * Date	12,456			0.64		0.64	
All factors	12,400	126	<0.001	0.67	7.9	0.67	8.5
*Relative adult bee models*							
Region * Date	4,829			0.61		0.61	
All factors	4,818	40	<0.001	0.64	7.2	0.64	7.2

Relative sealed brood count means that the dependent variable is the sealed brood count, expressed as a percentage of the adult bee count of the same colony on the same date. Relative adult bee count means that the adult bee count has been expressed as a percentage of the adult bee count of the same colony on the previous date. Models included 2-factor interactions of fixed effects, colony as a random effect, and autoregressive correlations. The “all-factors” models shown have had nonsignificant interactions removed and are the same as the corresponding models used for effect estimates, but to allow comparisons between models with different fixed effects are here presented as maximum likelihood models.

^a^Akaike Information Criterion.

^b^See Nakagawa and Schielzeth (2013). *R*^2^_(m)_ gives the proportion of the variance which is explained by the fixed effects of a mixed model. *R*^2^_(c)_ gives the proportion of the variance which is explained by the total model.

^c^Equals (100 * (*R*^2^(indicated model) − *R*^2^(region & date model))/(1 − *R*^2^(region & date model)).

Fumagillin treatment did not affect the ratio of brood to bees (*P* > 0.05). Fumagillin was associated with a significant main effect on relative adult bee counts (*F* = 4.032, df = 1, 294, *P* = 0.046), but the effect estimate was not significant (1.4 ± 2.1%; *t* = 0.66, df = 294, *P* = 0.51) when the dates during summer 2014, preceding the first fumagillin application, were excluded (statistical Supplementary file S1 - Table 6.7).

Visible disease did not affect the ratio of brood to bees (*P* > 0.05). Visible disease affected the relative adult population as an interaction with date (*F* = 3.6, df = 4, 745, *P* = 0.007) but was not significant by contrast at any individual date. At the May 2015, June 2015, and May 2016 inspections, colonies that had previous visible disease symptoms were (insignificantly) smaller. At the August inspections in both years, colonies that had been diseased in June were insignificantly larger, as a percent of their June populations, than those which had been healthy.

**Fig. 4.**
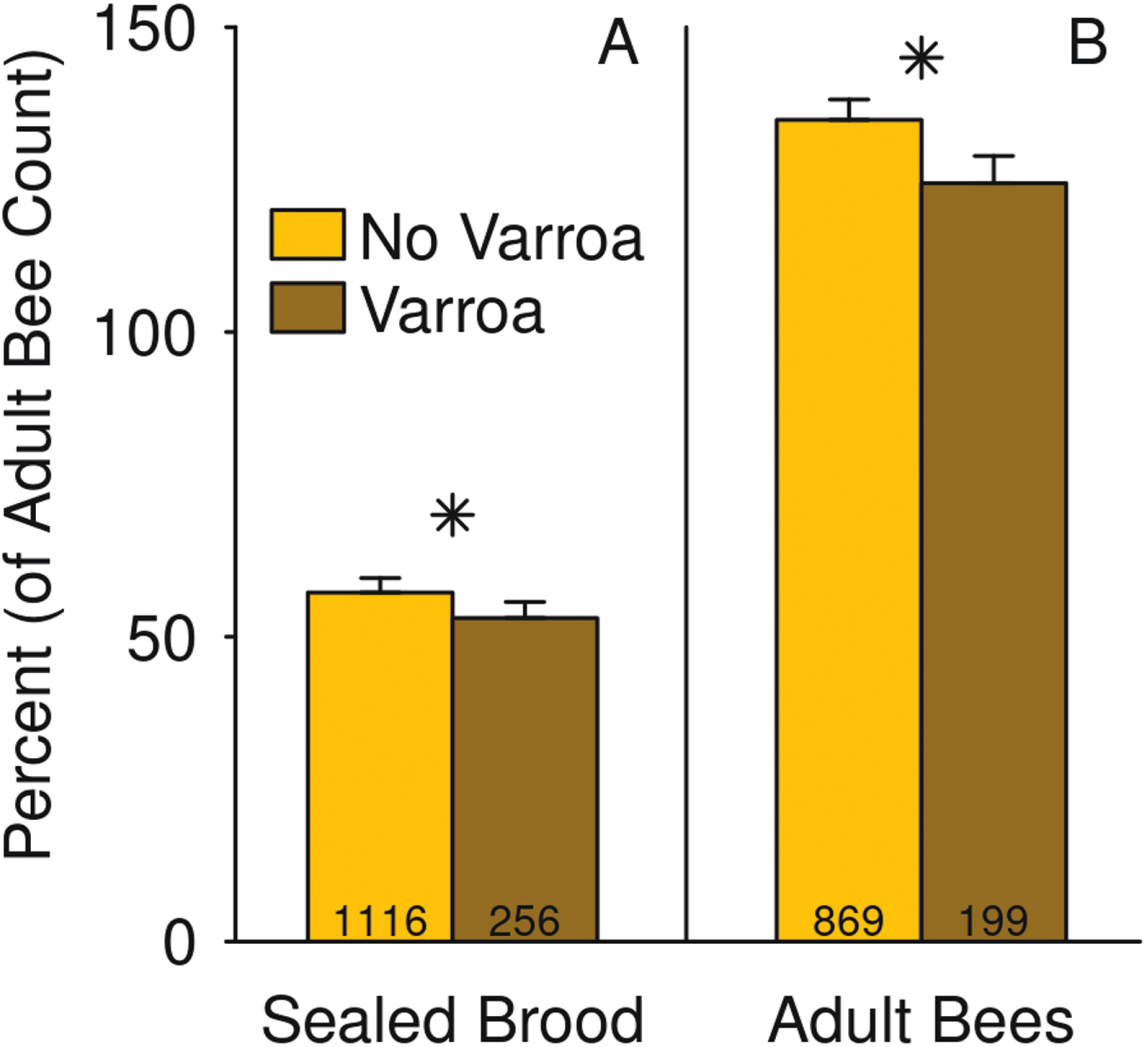
Colonies with detectable varroa had a lower rate of brood production relative to their size, and were smaller, relative to their prior size, at the next inspection. A) Sealed brood count expressed as a percent of adult bee count on the date of the varroa detection, and B) adult bee count on the subsequent inspection date, expressed as a percent of the adult bee count on the date of the varroa detection. Emmeans ± SE are shown, as estimated from the “all-factors” models, averaged across dates and regions. Stars indicate a statistically significant difference (*P* < 0.05) between the emmeans of colonies with and without varroa detections. The number of colony inspections (A) or pairs of colony inspections (B) used for estimates of varroa-detected and not-detected colonies are indicated at the bottoms of the bars.

Varroa detection was associated with a lower ratio of brood to bees on the date of the detection (Fig. 4A; main effect size: −4.2 ± 2.1 %; *F* = 6.9, df = 1, 1004, *P* = 0.009; *t* = −2.0, df = 1004, *P* = 0.042). There was also a significant interaction between varroa and region. Within region, the effect of varroa was significant only in SAB (effect size: −8.9 ± 3.3%; *t* = *−*2.6, df = 1004, *P* = 0.009). Similarly, varroa detections were associated with smaller adult bee populations at the subsequent assessment date (Fig. 4B; estimated difference: −10.4 ± 4.5% of the colony size on the first date; *t* = −2.3, df = 294, *P* = 0.023).

**Fig. 5.**
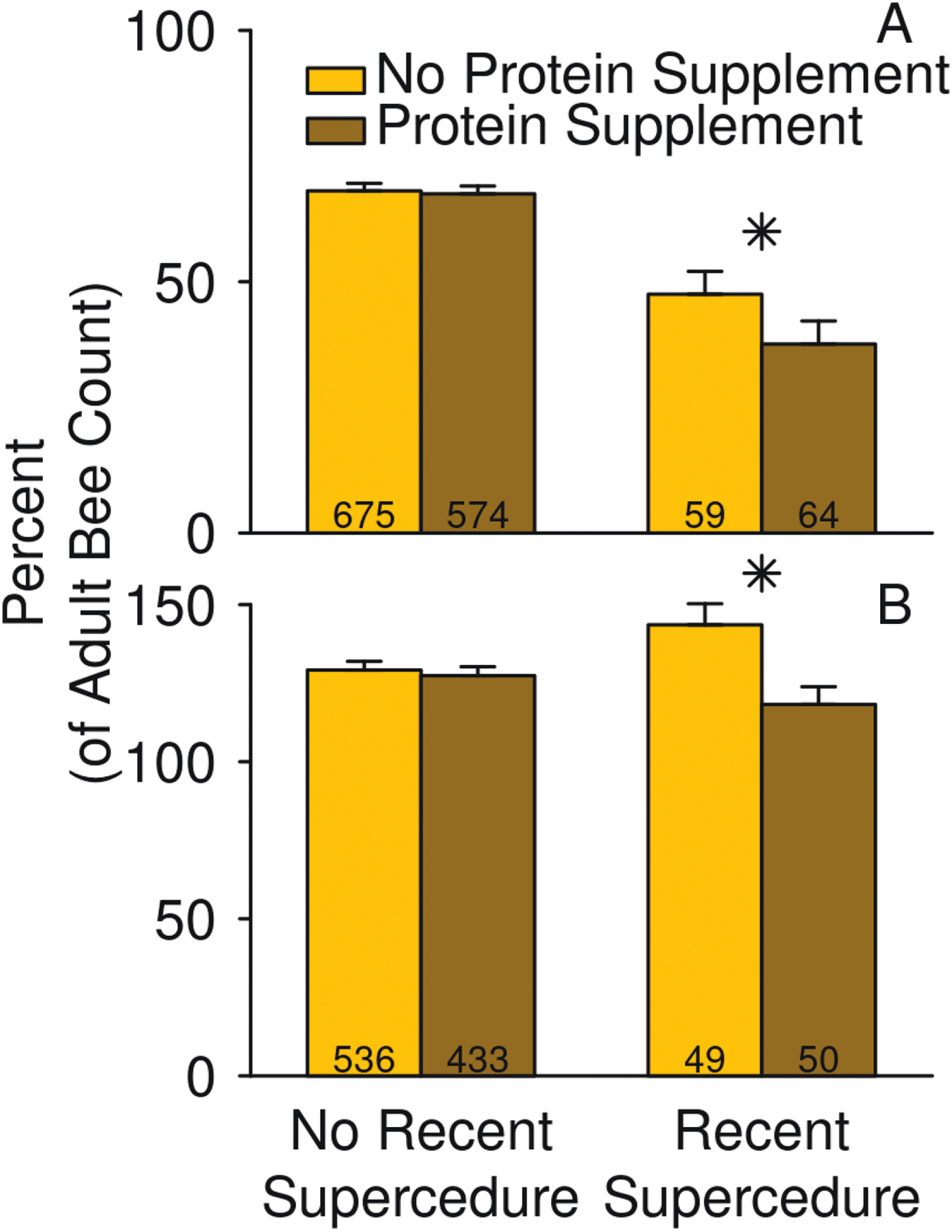
Protein supplements were only associated with reduced colony size in colonies that experienced recent supersedure: A) sealed brood count expressed as a percent of adult bee count on the date at which the queen event was detected, and B) adult bee count on the subsequent inspection date, expressed as a percent of the adult bee count on the date of the queen inspection. Emmeans ± SE are shown, as estimated from the “all-factors” models, averaged across regions and dates. Stars indicate a statistically significant difference (*P* < 0.05) between the emmeans of colonies that received protein supplements and those that did not. The number of colony inspections (A) or pairs of colony inspections (B) used for estimates are indicated at the bottoms of the bars.

Protein supplements unexpectedly reduced the ratio of brood to bees in a way which varied among regions and dates (Patties * Region * Date: *F* = 2.21, df = 10, 1004, *P* = 0.015). There was also an interaction between protein supplements and queen events (Fig. 5A; *F* = 6.6, df = 1, 1004, *P* = 0.010). Protein supplements did not affect the ratio of brood to bees in colonies which had not had a recent queen event (effect size: −0.5 ± 2.0%; *t* = −0.32, df = 315, *P* = 0.75), but were associated with less sealed brood in colonies that had superseded (effect size: −9.9 ± 3.6%; *t* = −2.7, df = 315, *P* = 0.007). Similarly, protein supplementation did not affect relative adult bee counts in colonies that had been queenright at the previous inspection (Fig. 5B; effect size: −1.7 ± 2.2%; *t* = −0.81, df = 294, *P* = 0.42) but produced smaller adult bee counts in colonies that had recently superseded (Fig. 5B; effect size: −25.3 ± 7.6 %, *t* = −3.3, df = 294, *P* = 0.001).

### Honey Production

Most of the variability in honey production (85%) could be attributed to region and date (Table 5), but the addition of treatments and field-observed events increased the proportion of variability explained and, in most cases, resulted in significant improvements (reduced AIC and *P* < 0.05; Table 5). For honey production, we distinguished events that were detected before the main honey flow (June inspections) from those detected after the honey flow (August inspections) and tested both. Fixed effects of the all-factors model accounted for 90% of the variability in the honey production dataset, which represents a 28% reduction in the amount of unexplained variability compared to the reference model.

**Table 5.** Comparison of maximum likelihood models of honey production

Fixed effects	AIC^a^	Likelihood ratio	*P*	Coefficient of determination *R*^2^_(m)_^b^	Percent reduction in unexplained variability (fixed effects)^c^	Coefficient of determination *R*^2^_(c)_^b^	Percent reduction in unexplained variability (total)^c^
Region & Date	3,534			0.8539		0.9314	
Region & Date + preVarroa	3,539	2.345	0.67	0.8546	0.48	0.9298	−2.275
Region & Date + Varroa	3,529	12.62	0.013	0.8558	1.27	0.9387	10.62
Region & Date + preSick	3,524	17.65	0.001	0.8692	10.47	0.9423	15.96
Region & Date + Disease	3,528	14.06	0.007	0.8685	10.00	0.9342	4.109
Region & Date + preQueen	3,525	17.25	0.002	0.8596	3.915	0.9347	4.875
Region & Date + Queen Event	3,531	10.47	0.033	0.8636	6.629	0.9433	17.29
Region & Date + Treatments	3,538	13.57	0.14	0.8591	3.529	0.9335	3.069
All factors	3,491	78.73	<0.001	0.8951	28.2	0.9684	53.98

Models included 2-factor interactions of fixed effects, colony as a random effect, and autoregressive correlations. The model with treatments is the optimized model presented in Peirson et al. (2024), but for comparison with the other models is run as a maximum likelihood model using the reduced dataset which arises from excluding missing observations of varroa and disease. The “all-factors” model shown has had nonsignificant interactions removed and is the same as the corresponding models used for effect estimates, but to allow comparisons between models with different fixed effects it is here treated as a maximum likelihood model, rather than the restricted maximum likelihood model used to measure factor significance and estimate effect sizes. “pre” in the factor names indicates observations (of varroa, disease, supersedure) taken before the honey flow (ie in June); otherwise the observations are from the end of the honey flow (August inspections).

^a^Akaike Information Criterion.

^b^See Nakagawa and Schielzeth (2013). *R*^2^(m) gives the proportion of the variance which is explained by the fixed effects of a mixed model. *R*^2^_(c)_ gives the proportion of the variance which is explained by the total model.

^c^Equals (100 * (*R*^2^(indicated model) − *R*^2^(region & date model))/(1 − *R*^2^(region & date model)).

**Fig. 6.**
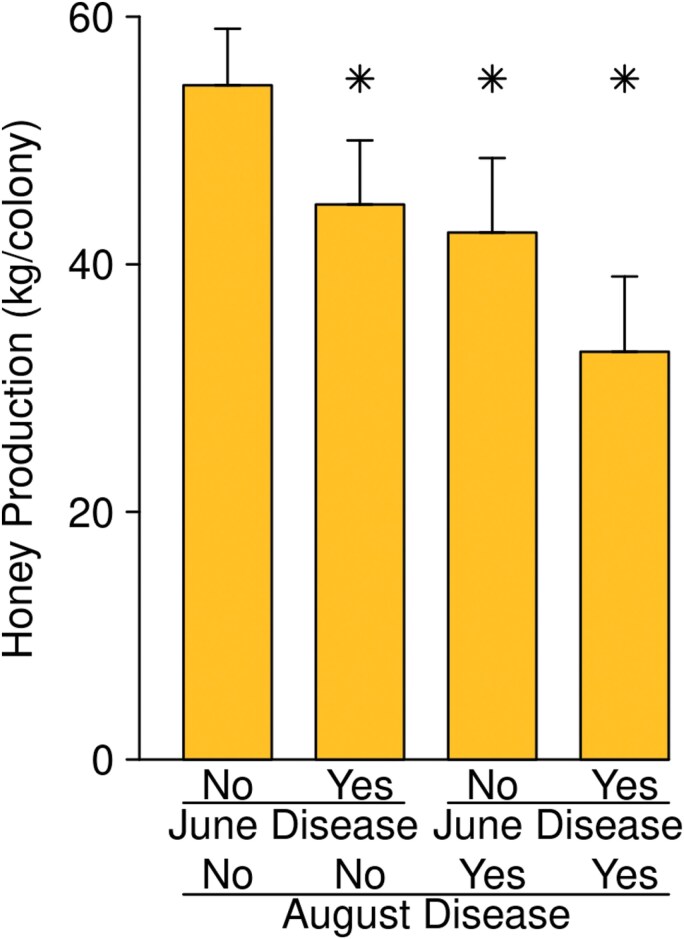
Detection of visible disease was associated with large reductions in honey production. Only Alberta colonies were used for honey production; consequently, most cases of disease were cases of chalkbrood. Emmeans ± SE are shown, as estimated from the “all-factors” model, averaged across regions and years. Stars illustrate that a bar is statistically different (*P* < 0.05) from the case where no disease was detected (leftmost bar). As modeled, disease detected in June and disease detected in August are additive main effects, not an interaction; consequently, the number of cases used to estimate the effects do not correspond to the bars. Number of cases used for model estimates: June, healthy: 331; June, sick: 46; August, healthy: 349; August, sick: 18.

**Fig. 7.**
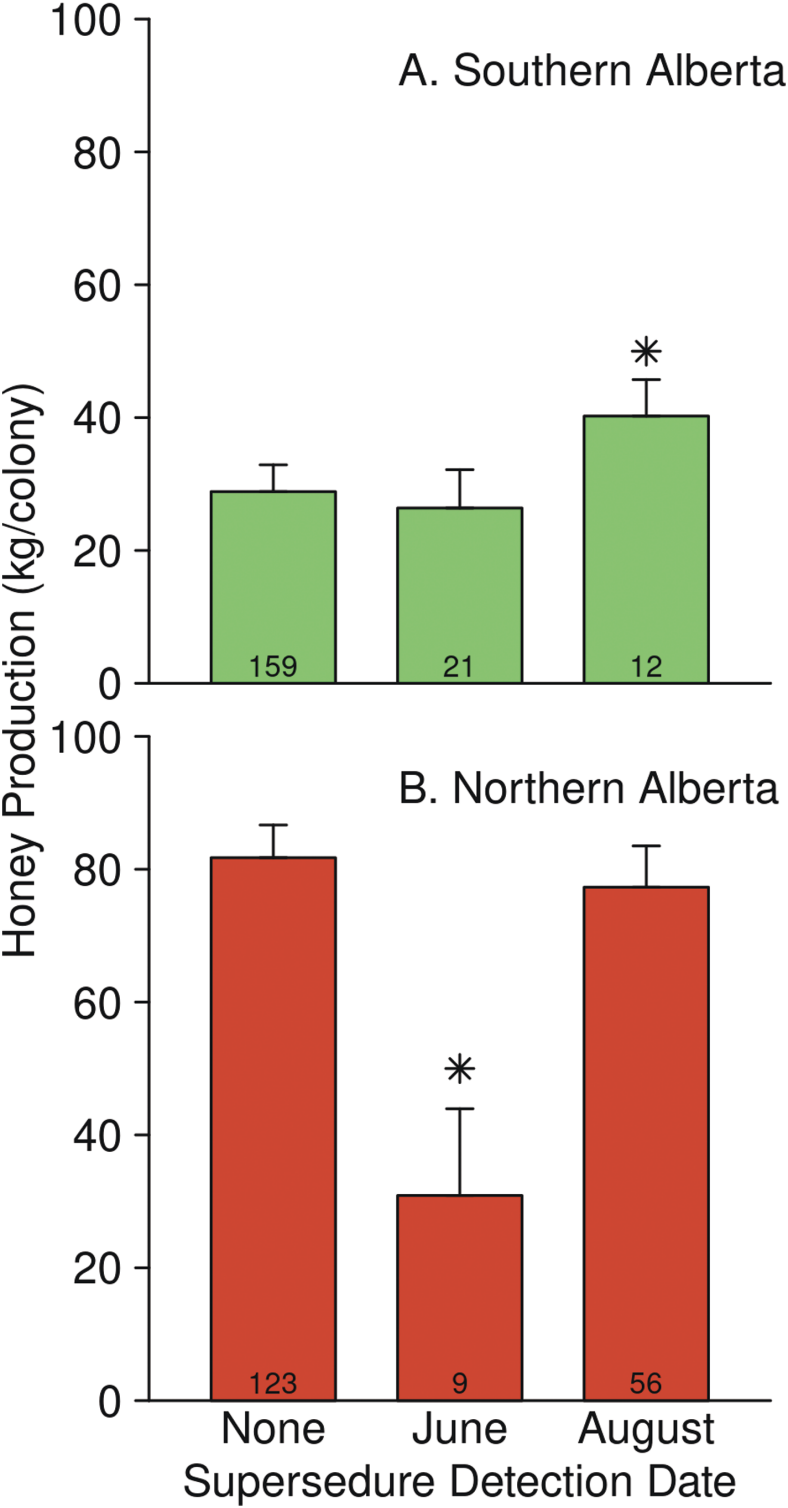
The effect of supersedure on honey production depended on region and date. Emmeans ± SE are shown, as estimated from the “all-factors” model, averaged across year. None: colonies that did not supersede; June: queen event detected at the June inspection; August: queen event detected at the August inspection (ie the colony superseded during the honey flow). Stars indicate a statistically significant difference (*P* < 0.05) between the emmeans of colonies that superseded and those that did not.

**Fig. 8.**
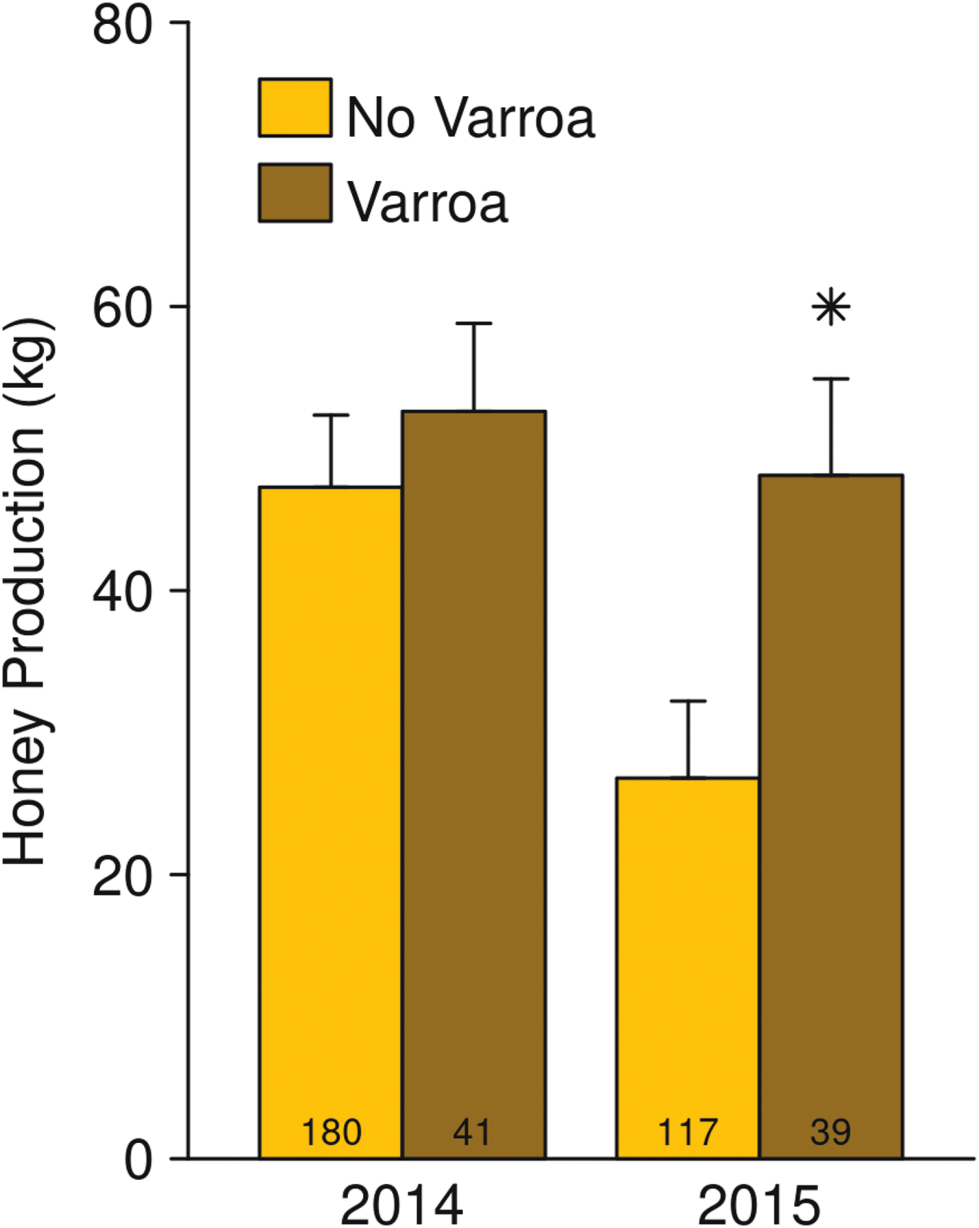
Varroa detection in August was associated with higher honey production. Emmeans ± SE are shown, as estimated from the “all-factors” models, averaged across regions. Stars indicate a statistically significant difference (*P* < 0.05) between the honey production of colonies with and without detected varroa in August of the same year.

There was no interaction between either treatment and any of the field observations. Visible disease detected before the honey flow and visible disease detected after the honey flow were both significant main effects (before: *F* = 13.12, df = 1, 141, *P* < 0.001; after: *F* = 9.264, df = 1, 141, *P* = 0.003). Visible disease (Fig. 6) observed before the honey flow reduced honey production by 9.6 ± 2.7 kg (*t* = −3.622, df = 141, *P* < 0.001) and visible disease observed after the honey flow reduced honey production by 11.9 ± 3.9 kg (*t* = −3.044, df = 141, *P* = 0.003). Queen supersedures which occurred before the honey flow caused very large reductions in honey production in NAB but not in SAB (Fig. 7; NAB: −51 ± 13 kg; *t* = −4.024, df = 141, *P* < 0.001; SAB: −2.5 ± 4.5 kg, *t* = −0.5441, df = 141, *P* = 0.58). Supersedures detected at the end of the honey flow were associated with higher honey production in SAB but not in NAB (NAB: −4.4 ± 5.9 kg; *t* = −0.7492, df = 141, *P* = 0.45; SAB: 11.4 ± 4.5 kg; *t* = 2.518, df = 141, *P* = 0.01293). Varroa, when detected before the honey flow, had an effect which was negative but not statistically significant (−6.1 ± 6.2 kg; *t* = −0.9811, df = 141, *P* = 0.33). When detected at the end of the honey flow (Fig. 8), varroa was associated with greater honey production in 2015 (21.3 ± 6.0 kg; *t* = 3.563, df = 141, *P* = 0.001).

### Colony Survival

Visible disease detection increased the risk of colony death in all regions (hazard ratio: 2.08 ± 0.80; *χ*^2^ = 5.3, df = 1, *P* = 0.021; *z* = 1.88, *P* = 0.061) but the effect was significant only in SAB (hazard ratio in SAB: 5.69 ± 2.82; *z* = 3.51; *P* < 0.001; regional interaction effect: *χ*^2^ = 7.6, df = 2, *P* = 0.02). Varroa detection and queen supersedures did not significantly alter the risk of colony death.

### Prince Edward Island

In PEI, the greater range of mite counts allowed us to use mites per hundred bees, instead of mite detection, as a predictor. This additional analysis showed several interesting effects. Notably, each mite per hundred bees increased the probability of colony death by 4.7% (ratio = 1.047; *χ*^2^ = 5.4, df = 1, *P* = 0.020; *z* = 2.56, *P* = 0.010). The effect of mites on adult bee counts depended on fumagillin treatment (*F* = 6.8, df = 1, 166, *P* = 0.001) and date (*F* = 5.1, df = 5,166, *P* < 0.001), but only the effect in May 2015 was significant (effect of each mite per hundred bees: −373 bees; t = -4.79, df = 166, *P* < 0.001). Each mite per hundred bees also reduced the absolute sealed brood count by 160 (*F* = 18.6, df = 1, 172, *P* < 0.001; *t* = 4.3, df = 172, *P* < 0.001), the sealed brood count as a percentage of adult bees by 1% of the adult bee count (*F* = 15.63, df = 1, 172, *P* < 0.001; *t* = −3.81, df = 172, *P* < 0.001), and the subsequent adult bee count by 4.2% of the current adult bee count (*F* = 4.608, df = 1, 107, *P* = 0.034; *t* = −2.1, df = 72, *P* = 0.040).

## Discussion

Do beekeepers, from observing their own colonies, have the information they need to know which colonies will be successful? Beekeeper-observable events (varroa detection, disease detection, and supersedure) and beekeeper-applied treatments (protein supplements and fumagillin) had significant and large effects on colony size, honey production, and survival. However, varroa, disease, and supersedure are often overlooked and their effects are not all immediate. Even when carefully observed, they accounted for a small proportion of the total variability among colonies. Consequently, beekeepers are unlikely to recognize the true relationships between treatments or field observations, and outcomes.

The 3 event types and 2 treatments each accounted for single digit percentages of the variance among colonies at the same region and date, suggesting that all factors are about equally important. However, the 3 event variables differed in frequency and severity among regions and dates, and this portion of their effect is not part of our estimate of variance explained because it is captured by the reference (region and date) model. Therefore, the true effects of the field-observed factors are probably larger than estimated here, and larger than the effects of protein supplements and fumagillin. Smart et al. (2018) considered differences among apiaries and found that a model that accounted for varroa and disease was better than models based on land use alone.

*Varroa destructor* is the most serious current threat to honey bee colonies (Guzmán-Novoa et al. 2010). The 3 beekeeping operations we examined had varroa levels below those reported in surveys of commercial beekeepers (Traynor et al. 2016, NBDC 2017), and mostly below published treatment thresholds (Delaplane and Hood 1999, Strange and Sheppard 2001, Gatien and Currie 2003, Currie and Gatien 2006, Currie 2008), although recommended fall thresholds for varroa in western Canada have recently been lowered (Morfin et al. 2024). Nevertheless, the operations differed considerably in the severity and prevalence of varroa infestations. Outside PEI, samples containing more than one mite were rare, and this regional difference presumably contributed to the high rate of colony death at PEI. Varroa detections were also associated with small colony size in PEI, but not in Alberta. However, a significant effect of varroa on colony size could be detected across all regions and dates when brood production or subsequent colony size were expressed as a percent of the colony’s existing adult bee count. Varroa detection was associated with about 4% fewer sealed brood cells and subsequently about 10% fewer bees. Somewhat oddly, varroa detection in August was associated with higher honey yield, even though colonies with varroa were not larger. Perhaps some colonies were devoting more effort to foraging and less to brood rearing than their neighbors. This would lead to 3 effects: greater honey production, a higher proportion of phoretic (ie detectable) mites, and slower growth/faster population decline. Nevertheless, these observations suggest that varroa detection, even at levels well below published thresholds, predicts a slower population growth rate or faster decline than other colonies at the same site.

The most striking feature of our visible disease observations is the extent to which both the number and the type of visible diseases varied, and varied predictably, among operations. Colonies in NAB were nearly symptom-free throughout the experiment, likely because they were begun with package bees on largely irradiation-disinfected hive equipment. A single disease, chalkbrood, predominated in Southern Alberta, while diverse symptoms were observed at the blueberry pollinator in PEI. High frequencies of disease, particularly EFB, have been reported among beekeeping operations that engage in blueberry pollination (vanEngelsdorp et al. 2013, Grant et al. 2021, Fowler et al. 2023, McAfee et al. 2024).

Surveys of colony health often report rather low prevalences of visible diseases (NBDC 2017), but even nonhygienic colonies rapidly remove dead brood (Spivak and Danka 2021) and diseases can be difficult to detect reliably, even for trained inspectors (Stephan 2020). Furthermore, the task is very large. The average colony in this study had about 8,500 sealed brood cells as well as open and empty cells in the brood area. An inspector assigned to check 25 single brood chamber Langstroth colonies (each with ~17,000 potentially symptomatic cells) would have 425,000 cells to examine, not considering other tasks. Because comprehensive inspections are impossible, inspectors normally examine only a few frames per colony (Government of Alberta 2025) and it can be assumed that many light (<10 individuals/colony) and some moderate (<100 individuals/colony) infections are overlooked. In this dataset, 48% of colonies expressed visible disease symptoms at some time, but only 13% of colony inspections resulted in a disease detection, and nearly half of those cases involved ten or fewer detected individuals.

Visible diseases were associated with small effects on colony size, but large effects on honey production. The main disease found was chalkbrood, which is often regarded as a minor disease (Hedtke et al. 2011, OMAFRA 2015). However, the large effect on honey production, which has been reported previously (Aronstein and Murray 2010, van Haga et al. 2012, Desalegn 2015), belies that view. Seemingly, effort that would have gone into honey production was diverted to clear the infection and maintain colony size, resulting in a large economic loss for beekeepers. The very large effect of visible disease on the relative risk of colony death is harder to explain because chalkbrood is not known to affect adult bees. Perhaps chalkbrood served as a marker of some less visible stressor that was the true cause of colony death. Associations between chalkbrood symptoms and other pathogens, namely *V. destructor* and *Vairimorpha ceranae*, were previously reported (Hedtke et al. 2011). Similarly, vanEngelsdorp et al. (2013) reported that idiopathic brood disease syndrome, which also does not usually kill all the brood and has no known effects on adults, was a strong predictor of colony death.

Honey bee queens were previously reported to live for up to 5 yr (Szabo 1993), but several authors (Rangel and Tarpy 2016, Amiri et al. 2017, Traynor et al. 2021) have suggested that elevated rates of queen loss are now common, and this has also been our experience. Taking the present dataset as an example, after 25 mo, only 55% (47 of 85) of surviving colonies in SAB, 21% (14 of 65) in NAB, and 23% (3 of 13) in PEI retained their original queen (Table S9 in Peirson et al. 2024). This compares poorly with a previous report that 64% of queens in NAB survived to 22 mo (Szabo 1993). Annual or bi-annual queen replacement by the beekeeper has been recommended as a means of preventing spontaneous queen loss (Szabo 1993). However, the value of introduced queens may be questioned if they cannot be relied upon to survive.

Naturally occurring queen events have been identified as a major predictor of colony death (vanEngelsdorp et al. 2013), and queen replacements have been reported to reduce the risk of death (Gray et al. 2020, 2023). Here, queen replacements did not influence the risk of colony death in the short term. These results are not contradictory. Our dataset consisted mainly of naturally occurring supersedures, and only successful supersedures were counted. Thus, while queen replacement is a high-risk event for a colony, and colonies that spontaneously lose their queen might be a self-selected higher risk group, a colony that succeeds in producing a new queen is not at elevated risk of death, compared to its neighbors, going forward.

The effects of fumagillin and protein supplements were previously described in Peirson et al. (2024) and were largely unchanged by the addition of field-observed events to the model. Here we found an unexpected interaction between protein supplements and queen events. Protein supplemented and unsupplemented colonies produced similar amounts of brood (relative to their adult bee counts) and grew at similar rates provided that a queen event did not take place. Following a supersedure, unsupplemented colonies increased in size, but supplemented colonies produced less brood and declined in size. We previously speculated that protein supplements harmed adult bees, perhaps by increasing the prevalence of nosema (Peirson et al. 2024). If so, one interpretation of this interaction is that brood rearing in supplemented colonies depended on a continuous supply of healthy young nurse bees, which was interrupted by the supersedure.

Although researchers agree that many factors influence the health of honey bee colonies, quantitative estimates of the role played by each factor are not common. Differences among regions and dates accounted for most of the variability in adult bee counts and honey production, and about half the variability in sealed brood counts. However, individual beekeepers manage colonies within particular regions and can observe only one date at a time. We found that cumulatively, 3 types of beekeeper-observable events and 2 beekeeper-applied treatments explained between 5% (adult bee counts) and 28% (honey production) of the variability among colonies at the same region and date. This implies that most differences among colonies could not be explained using the information available to a beekeeper and arose from causes that are difficult to detect, such as asymptomatic pathogens. Furthermore, even detectable causes can be hard to connect to their effects; for example chalkbrood in June evidently reduces honey yield, which will not be known until August. Consequently, beekeepers often will not know why their colonies performed poorly, and surveys of beekeepers’ opinions about colony losses can be expected to reach the wrong conclusions.

Although each field-observed event and beekeeper-applied treatment had only a small effect, those effects were significant. Varroa at levels well below published thresholds was followed by considerably reduced colony size. A reputedly minor disease, chalkbrood, resulted in a much smaller honey crop and increased risk of death. Temporary queen loss in early summer greatly reduced honey production at one location. Increased control of these factors should produce real benefits. Beekeepers should be advised that further increases in efforts to control the diseases and pests that they can detect are likely to be profitable.

## Supplementary material

Supplementary material is available at *Journal of Economic Entomology* online.

toaf094_suppl_Supplementary_Materials
