# Supplementary material for: Supersedure, mites, and visible disease in Apis mellifera (Hymenoptera: Apidae) colonies explain differences in productivity and survival, but the effects may be difficult to see
Source: J Econ Entomol. 2025 Jun 13;118(4):1463–74. doi: 10.1093/jee/toaf094 (PMC12397970; doi:10.1093/jee/toaf094)
Supplement: toaf094_suppl_Supplementary_Materials [file toaf094_suppl_supplementary_materials.zip › Supplemental File 1.html]

Statistical Analysis Supplemement for Bee Health Paper 2


# Statistical Analysis Supplemement for Bee Health Paper 2

Analysis with RStudio build 375 “Cranberry Hibiscus” for Ubuntu Jammy
R version 4.4.1

# **Contents**

1. Varroa Summary Statistics
2. Visible Disease Summary Statistics
3. Adult Bee Count Model Comparisons and Results
4. Sealed Brood Count Model Comparisons and Results
5. Relative Sealed Brood Count Model Comparisons and Results
6. Relative Adult Bee Count Model Comparisons and Results
7. Honey Production Model Comparisons and Results
8. Survival Model Results

# **1. Varroa Summary Statistics**

Summary of Findings:

1. The number of bees tested was very high and the number of mites
   detected was very low overall
2. There were significant differences among regions and dates, and was
   no significant difference related to fumagillin treatment.
3. There was an apparent significant difference among protein
   treatments which was almost certainly an artefact of a small number of
   high-mite colonies being randomly assigned to the protein +
   treatment.
4. Mite count measurements appeared to follow a distribution that is
   very far from Normal. Occasional very high counts are likely to occur
   even when typical counts are very low.

1.1 Histogram of number of bees washed for varroa in the whole
experiment

#### **1.2 Bees Washed for Varroa**

| Samples Washed | Total Bees Washed | Bees per Sample | Standard Deviation | Smallest Sample | Largest Sample |
| --- | --- | --- | --- | --- | --- |
| 2112 | 452278 | 214.1 | 60.19 | 12 | 507.4 |

The number of samples in table 1.2 does not match the tables of
varroa counts because bees were not counted from eight samples, all of
which had zero mites.

#### **1.3 Varroa mites detected, by region**

| Region | Samples Washed | Sum of Mites | Mean of Mites per Sample | Standard Deviation of Mites per Sample | Median Mites per Sample | Smallest Count | Largest Count |
| --- | --- | --- | --- | --- | --- | --- | --- |
| Southern Alberta | 920 | 31 | 0.0337 | 0.2032 | 0 | 0 | 2 |
| Northern Alberta | 834 | 142 | 0.1703 | 0.5432 | 0 | 0 | 5 |
| Prince Edward Island | 366 | 1074 | 2.934 | 5.891 | 1 | 0 | 49 |

#### **1.4 Varroa per hundred bees, by region**

| Region | Samples Washed | Mean of Mites per Hundred Bees | Standard Deviation of Mites per Hundred Bees | Median Mites per Hundred Bees | Smallest Percent | Largest Percent |
| --- | --- | --- | --- | --- | --- | --- |
| Southern Alberta | 920 | 0.02319 | 0.1694 | 0 | 0 | 3.122 |
| Northern Alberta | 834 | 0.07748 | 0.2548 | 0 | 0 | 2.5 |
| Prince Edward Island | 366 | 1.745 | 3.981 | 0.5 | 0 | 33.33 |

#### **1.5 T test for differences in mites per hundred bees between NAB & SAB**

Welch Two Sample t-test:
`BHP$Varroa.Percent[BHP$Region == "Southern Alberta" & BHP$Viable == "Viable"]`
and
`BHP$Varroa.Percent[BHP$Region == "Northern Alberta" & BHP$Viable == "Viable"]`


| Test statistic | df | P value | Alternative hypothesis | mean of x | mean of y |
| --- | --- | --- | --- | --- | --- |
| -5.199 | 1426 | 2.297e-07 \* | two.sided | 0.02319 | 0.07748 |

#### **1.6 T test for differences in mites per hundred bees between SAB & PEI**

Welch Two Sample t-test:
`BHP$Varroa.Percent[BHP$Region == "Southern Alberta" & BHP$Viable == "Viable"]`
and
`BHP$Varroa.Percent[BHP$Region == "Prince Edward Island" & BHP$Viable == "Viable"]`


| Test statistic | df | P value | Alternative hypothesis | mean of x | mean of y |
| --- | --- | --- | --- | --- | --- |
| -8.271 | 365.5 | 2.493e-15 \* | two.sided | 0.02319 | 1.745 |

#### **1.7 T test for differences in mites per hundred bees between PEI & NAB**

Welch Two Sample t-test:
`BHP$Varroa.Percent[BHP$Region == "Prince Edward Island" & BHP$Viable == "Viable"]`
and
`BHP$Varroa.Percent[BHP$Region == "Northern Alberta" & BHP$Viable == "Viable"]`


| Test statistic | df | P value | Alternative hypothesis | mean of x | mean of y |
| --- | --- | --- | --- | --- | --- |
| 8.006 | 366.3 | 1.583e-14 \* | two.sided | 1.745 | 0.07748 |

#### **1.8 Mites per hundred bees, by date**

| Date | Samples Washed | Mean of Mites per Hundred Bees | Standard Deviation of Mites per Hundred Bees | Median Mites per Hundred Bees | Smallest Sample | Largest Sample |
| --- | --- | --- | --- | --- | --- | --- |
| May 2014 | 239 | 0.002092 | 0.03234 | 0 | 0 | 0.5 |
| June 2014 | 316 | 0.5562 | 1.91 | 0 | 0 | 23.49 |
| August 2014 | 299 | 0.1476 | 0.3894 | 0 | 0 | 3.797 |
| November 2014 | 281 | 0.3768 | 1.509 | 0 | 0 | 20 |
| May 2015 | 245 | 1.129 | 4.186 | 0 | 0 | 33.33 |
| June 2015 | 192 | 0.3523 | 1.271 | 0 | 0 | 9.747 |
| August 2015 | 200 | 0.1768 | 0.4273 | 0 | 0 | 3.122 |
| November 2015 | 193 | 0.07313 | 0.4484 | 0 | 0 | 4.46 |
| May 2016 | 155 | 0.02925 | 0.1781 | 0 | 0 | 1.605 |

#### **1.9 Unadjusted P values of pairwise T tests for mites per hundred bees, by date**

|  | May 2014 | June 2014 | August 2014 | November 2014 | May 2015 | June 2015 | August 2015 | November 2015 |
| --- | --- | --- | --- | --- | --- | --- | --- | --- |
| June 2014 | 4.448e-07 | NA | NA | NA | NA | NA | NA | NA |
| August 2014 | 4.89e-10 | 0.0002302 | NA | NA | NA | NA | NA | NA |
| November 2014 | 4.202e-05 | 0.201 | 0.01401 | NA | NA | NA | NA | NA |
| May 2015 | 3.535e-05 | 0.04769 | 0.0003115 | 0.008093 | NA | NA | NA | NA |
| June 2015 | 0.0001812 | 0.1495 | 0.03119 | 0.8491 | 0.006371 | NA | NA | NA |
| August 2015 | 2.967e-08 | 0.0007501 | 0.4377 | 0.03591 | 0.0004801 | 0.07036 | NA | NA |
| November 2015 | 0.02928 | 2.129e-05 | 0.05939 | 0.001627 | 0.0001143 | 0.004444 | 0.0195 | NA |
| May 2016 | 0.06208 | 1.81e-06 | 1.161e-05 | 0.000167 | 5.472e-05 | 0.0006122 | 1.444e-05 | 0.2151 |

#### **1.10 Mites per hundred bees, by treatment group**

| Patties | Fumagillin | Samples Washed | Mean of Mites per Hundred Bees | Standard Deviation of Mites per Hundred Bees | Median Mites per Hundred Bees | Smallest Sample | Largest Sample |
| --- | --- | --- | --- | --- | --- | --- | --- |
| No | No | 544 | 0.222 | 0.9274 | 0 | 0 | 13.64 |
| No | Yes | 578 | 0.3086 | 1.59 | 0 | 0 | 33.33 |
| Yes | No | 463 | 0.3924 | 2.006 | 0 | 0 | 24.38 |
| Yes | Yes | 535 | 0.4556 | 2.344 | 0 | 0 | 33.33 |

#### **1.11 T test for differences in mites per hundred bees between fumagillin treatment groups**

Welch Two Sample t-test:
`BHP$Varroa.Percent[BHP$Fumagillin == "Yes" & BHP$Viable == "Viable"]`
and
`BHP$Varroa.Percent[BHP$Fumagillin == "No" & BHP$Viable == "Viable"]`


| Test statistic | df | P value | Alternative hypothesis | mean of x | mean of y |
| --- | --- | --- | --- | --- | --- |
| 1.031 | 2063 | 0.3029 | two.sided | 0.3792 | 0.3004 |

#### **1.12 T test for differences in mites per hundred bees between protein treatment groups**

Welch Two Sample t-test:
`BHP$Varroa.Percent[BHP$Patties == "Yes" & BHP$Viable == "Viable"]`
and
`BHP$Varroa.Percent[BHP$Patties == "No" & BHP$Viable == "Viable"]`


| Test statistic | df | P value | Alternative hypothesis | mean of x | mean of y |
| --- | --- | --- | --- | --- | --- |
| 2.004 | 1589 | 0.04525 \* | two.sided | 0.4263 | 0.2666 |

### **Investigation of significant protein effect:**

#### **1.13 Mites per hundred bees, by region and protein treatment**

| Region | Patties | Samples Washed | Mean of Mites per Hundred Bees | Standard Deviation of Mites per Hundred Bees | Median Mites per Hundred Bees | Smallest Sample | Largest Sample |
| --- | --- | --- | --- | --- | --- | --- | --- |
| Southern Alberta | No | 462 | 0.01841 | 0.1205 | 0 | 0 | 1.538 |
| Southern Alberta | Yes | 458 | 0.02802 | 0.2074 | 0 | 0 | 3.122 |
| Northern Alberta | No | 471 | 0.08833 | 0.2929 | 0 | 0 | 2.5 |
| Northern Alberta | Yes | 363 | 0.0634 | 0.1942 | 0 | 0 | 1.5 |
| Prince Edward Island | No | 189 | 1.318 | 2.943 | 0.5 | 0 | 33.33 |
| Prince Edward Island | Yes | 177 | 2.201 | 4.817 | 0.5 | 0 | 33.33 |

#### **1.14.1 T test for differences in mites per hundred bees between protein treatment groups (Northern Alberta)**

Welch Two Sample t-test:
`BHP$Varroa.Percent[BHP$Region == "Northern Alberta" & BHP$Patties == "Yes" & BHP$Viable == "Viable"]`
and
`BHP$Varroa.Percent[BHP$Region == "Northern Alberta" & BHP$Patties == "No" & BHP$Viable == "Viable"]`


| Test statistic | df | P value | Alternative hypothesis | mean of x | mean of y |
| --- | --- | --- | --- | --- | --- |
| -1.474 | 814.8 | 0.1408 | two.sided | 0.0634 | 0.08833 |

#### **1.14.2 T test for differences in mites per hundred bees between protein treatment groups (Southern Alberta)**

Welch Two Sample t-test:
`BHP$Varroa.Percent[BHP$Region == "Southern Alberta" & BHP$Patties == "Yes" & BHP$Viable == "Viable"]`
and
`BHP$Varroa.Percent[BHP$Region == "Southern Alberta" & BHP$Patties == "No" & BHP$Viable == "Viable"]`


| Test statistic | df | P value | Alternative hypothesis | mean of x | mean of y |
| --- | --- | --- | --- | --- | --- |
| 0.8588 | 732.8 | 0.3907 | two.sided | 0.02802 | 0.01841 |

#### **1.14.3 T test for differences in mites per hundred bees between protein treatment groups (Prince Edward Island)**

Welch Two Sample t-test:
`BHP$Varroa.Percent[BHP$Region == "Prince Edward Island" & BHP$Patties == "Yes" & BHP$Viable == "Viable"]`
and
`BHP$Varroa.Percent[BHP$Region == "Prince Edward Island" & BHP$Patties == "No" & BHP$Viable == "Viable"]`


| Test statistic | df | P value | Alternative hypothesis | mean of x | mean of y |
| --- | --- | --- | --- | --- | --- |
| 2.101 | 287.7 | 0.03653 \* | two.sided | 2.201 | 1.318 |

**Conclusion:** The significant difference in average
mite load of protein supplemented and un-supplemented colonies arose
from Prince Edward Island colonies and was not apparent in colonies at
the other two locations.

#### **1.15 Number of mite-positive dates per colony, by region**

|  | 0 | 1 | 2 | 3 | 4 | 5 | 6 |
| --- | --- | --- | --- | --- | --- | --- | --- |
| Southern Alberta | 92 | 23 | 2 | 0 | 0 | 0 | 0 |
| Northern Alberta | 45 | 55 | 18 | 4 | 1 | 0 | 0 |
| PEI | 4 | 5 | 24 | 19 | 16 | 6 | 2 |

#### **1.16 Number of mite-positive dates per colony by fumagillin treatment**

|  | 0 | 1 | 2 | 3 | 4 | 5 | 6 |
| --- | --- | --- | --- | --- | --- | --- | --- |
| fumeyes | 70 | 42 | 21 | 14 | 8 | 4 | 1 |
| fumeno | 71 | 41 | 23 | 9 | 9 | 2 | 1 |

#### **1.17 Number of mite-positive dates per colony by protein treatment**

|  | 0 | 1 | 2 | 3 | 4 | 5 | 6 |
| --- | --- | --- | --- | --- | --- | --- | --- |
| pattyyes | 74 | 41 | 19 | 13 | 5 | 3 | 2 |
| pattyno | 67 | 42 | 25 | 10 | 12 | 3 | 0 |

**Conclusion:** Neither treatment affected the overall
frequency of mite detection.

1.18 Representative histograms of mite counts among colonies,
illustrating that the distribution was not Gaussian (Normal). Mite-free
samples, which were the overwhelming majority, are not shown.

1.19 Boxplots of Mite Counts by Date and Patty Treatment in PEI. Median
mite counts were similar in the two groups, but the ‘Patties’ group had
a few additional high count outliers in 2014.

# **2 Visible Disease Summary Statistics**

#### **2.1 Disease detections per inspection, whole experiment**

| Number of Diseases | Number of Inspections |
| --- | --- |
| 0 | 1842 |
| 1 | 241 |
| 2 | 26 |
| 3 | 3 |

#### **2.2 Disease detections per inspection, by region**

| Region | Number of Diseases | Number of Inspections |
| --- | --- | --- |
| Southern Alberta | 0 | 829 |
| Southern Alberta | 1 | 116 |
| Southern Alberta | 2 | 3 |
| Northern Alberta | 0 | 822 |
| Northern Alberta | 1 | 20 |
| Prince Edward Island | 0 | 191 |
| Prince Edward Island | 1 | 105 |
| Prince Edward Island | 2 | 23 |
| Prince Edward Island | 3 | 3 |

#### **2.3.0 Disease detections by region (binary)**

|  | Disease Detected | No Disease Detected |
| --- | --- | --- |
| SAB | 119 | 829 |
| NAB | 20 | 822 |
| PEI | 131 | 191 |

#### **2.3.1 Chi squared test for differences among regions**

Pearson’s Chi-squared test: `diseases`


| Test statistic | df | P value |
| --- | --- | --- |
| 306.7 | 2 | 2.58e-67 \* |

#### **2.3.2 Chi squared test for difference between NAB & SAB**

Pearson’s Chi-squared test with Yates’ continuity correction:
`diseases[c(1, 2), ]`


| Test statistic | df | P value |
| --- | --- | --- |
| 63.08 | 1 | 1.988e-15 \* |

#### **2.3.3 Chi squared test for differences between SAB & PEI**

Pearson’s Chi-squared test with Yates’ continuity correction:
`diseases[c(1, 3), ]`


| Test statistic | df | P value |
| --- | --- | --- |
| 118.5 | 1 | 1.326e-27 \* |

#### **2.3.4 Chi squared test for differences between NAB & PEI**

Pearson’s Chi-squared test with Yates’ continuity correction:
`diseases[c(2, 3), ]`


| Test statistic | df | P value |
| --- | --- | --- |
| 299.4 | 1 | 4.483e-67 \* |

#### **2.4 Disease detections per inspection by date**

| Date | Number of Diseases | Number of Inspections |
| --- | --- | --- |
| May 2014 | 0 | 209 |
| May 2014 | 1 | 31 |
| June 2014 | 0 | 236 |
| June 2014 | 1 | 67 |
| June 2014 | 2 | 8 |
| August 2014 | 0 | 248 |
| August 2014 | 1 | 43 |
| August 2014 | 2 | 7 |
| November 2014 | 0 | 216 |
| November 2014 | 1 | 3 |
| May 2015 | 0 | 215 |
| May 2015 | 1 | 41 |
| May 2015 | 2 | 8 |
| May 2015 | 3 | 3 |
| June 2015 | 0 | 186 |
| June 2015 | 1 | 30 |
| June 2015 | 2 | 1 |
| August 2015 | 0 | 205 |
| August 2015 | 1 | 16 |
| August 2015 | 2 | 2 |
| November 2015 | 0 | 167 |
| November 2015 | 1 | 2 |
| May 2016 | 0 | 160 |
| May 2016 | 1 | 8 |

#### **2.5.0 Disease detections by date (binary)**

|  | Disease Detected | No Disease Detected |
| --- | --- | --- |
| May 2014 | 31 | 209 |
| June 2014 | 75 | 236 |
| August 2014 | 50 | 248 |
| November 2014 | 3 | 216 |
| May 2015 | 52 | 215 |
| June 2015 | 31 | 186 |
| August 2015 | 18 | 205 |
| November 2015 | 2 | 167 |
| May 2016 | 8 | 160 |

#### **2.5.1 Chi squared test for differences among dates**

Pearson’s Chi-squared test: `diseases`


| Test statistic | df | P value |
| --- | --- | --- |
| 111.4 | 8 | 1.988e-20 \* |

#### **2.6 Disease detections per inspection by Treatment Group**

| Patties | Fumagillin | Number of Diseases | Number of Inspections |
| --- | --- | --- | --- |
| No | No | 0 | 478 |
| No | No | 1 | 53 |
| No | No | 2 | 6 |
| No | Yes | 0 | 501 |
| No | Yes | 1 | 68 |
| No | Yes | 2 | 7 |
| No | Yes | 3 | 1 |
| Yes | No | 0 | 391 |
| Yes | No | 1 | 68 |
| Yes | No | 2 | 5 |
| Yes | No | 3 | 2 |
| Yes | Yes | 0 | 472 |
| Yes | Yes | 1 | 52 |
| Yes | Yes | 2 | 8 |

#### **2.7.0 Disease detections by treatment group (binary)**

|  | Disease Detected | No Disease Detected |
| --- | --- | --- |
| No Treatment | 59 | 478 |
| Fumagillin Only | 76 | 501 |
| Patties Only | 75 | 391 |
| Both Treatments | 60 | 472 |

#### **2.7.1 Chi squared test for differences among treatment groups**

Pearson’s Chi-squared test: `diseases`


| Test statistic | df | P value |
| --- | --- | --- |
| 7.295 | 3 | 0.06306 |

#### **2.7.2 Disease detections by protein treatment**

|  |  |  |
| --- | --- | --- |
| patties | 135 | 863 |
| nopatties | 135 | 979 |

#### **2.7.3 Chi squared test for effect of protein treatment**

Pearson’s Chi-squared test with Yates’ continuity correction:
`patties`


| Test statistic | df | P value |
| --- | --- | --- |
| 0.8146 | 1 | 0.3668 |

#### **2.7.4 Disease detections by fumagillin treatment**

|  |  |  |
| --- | --- | --- |
| fume | 136 | 973 |
| nofume | 134 | 869 |

#### **2.7.5 Chi squared test for effect of fumagillin**

Pearson’s Chi-squared test with Yates’ continuity correction:
`fume`


| Test statistic | df | P value |
| --- | --- | --- |
| 0.474 | 1 | 0.4912 |

#### **2.8.0 Disease detections per colony whole experiment**

| Disease.Count | n |
| --- | --- |
| 0 | 195 |
| 1 | 117 |
| 2 | 40 |
| 3 | 9 |

#### **2.9.0 Disease detections per colony by Region**

| Region | Disease.Count | n |
| --- | --- | --- |
| Southern Alberta | 0 | 50 |
| Southern Alberta | 1 | 59 |
| Southern Alberta | 2 | 8 |
| Northern Alberta | 0 | 107 |
| Northern Alberta | 1 | 15 |
| Northern Alberta | 2 | 1 |
| Prince Edward Island | 0 | 38 |
| Prince Edward Island | 1 | 43 |
| Prince Edward Island | 2 | 31 |
| Prince Edward Island | 3 | 9 |

#### **2.10.0 Disease detections per colony by Region (binary)**

|  | Disease Detected | No Disease Detected |
| --- | --- | --- |
| SAB | 67 | 50 |
| NAB | 16 | 107 |
| PEI | 83 | 38 |

#### **2.10.1 Chi squared test for differences in number of sick colonies among regions**

Pearson’s Chi-squared test: `diseases`


| Test statistic | df | P value |
| --- | --- | --- |
| 84.75 | 2 | 3.955e-19 \* |

#### **2.11 Disease detections per colony by Treatment Group**

| Fumagillin | Patties | Disease.Count | n |
| --- | --- | --- | --- |
| No | No | 0 | 50 |
| No | No | 1 | 26 |
| No | No | 2 | 10 |
| No | No | 3 | 2 |
| No | Yes | 0 | 43 |
| No | Yes | 1 | 36 |
| No | Yes | 2 | 7 |
| No | Yes | 3 | 3 |
| Yes | No | 0 | 42 |
| Yes | No | 1 | 31 |
| Yes | No | 2 | 15 |
| Yes | No | 3 | 1 |
| Yes | Yes | 0 | 60 |
| Yes | Yes | 1 | 24 |
| Yes | Yes | 2 | 8 |
| Yes | Yes | 3 | 3 |

#### **2.12.0 Disease detections per colony by Treatment Group (binary)**

|  | Disease Detected | No Disease Detected |
| --- | --- | --- |
| None | 38 | 50 |
| Protein Only | 46 | 43 |
| Fumagillin Only | 47 | 42 |
| Both | 35 | 60 |

#### **2.12.1 Chi squared test for differences among treatment groups**

Pearson’s Chi-squared test: `diseases`


| Test statistic | df | P value |
| --- | --- | --- |
| 6.308 | 3 | 0.09753 |

#### **2.12.2 Disease detections per colony by protein treatment**

|  |  |  |
| --- | --- | --- |
| patties | 82 | 102 |
| nopatties | 84 | 93 |

#### **2.12.3 Chi squared test for effect of patties**

Pearson’s Chi-squared test with Yates’ continuity correction:
`patties`


| Test statistic | df | P value |
| --- | --- | --- |
| 0.1986 | 1 | 0.6559 |

#### **2.12.4 Disease detections per colony by fumagillin treatment**

|  |  |  |
| --- | --- | --- |
| fume | 81 | 103 |
| nofume | 85 | 92 |

#### **2.12.5 Chi squared test for effect of fumagillin**

Pearson’s Chi-squared test with Yates’ continuity correction:
`fume`


| Test statistic | df | P value |
| --- | --- | --- |
| 0.4315 | 1 | 0.5113 |

#### **2.13 Categories of disease, whole experiment**

| Disease | Disease - Positive Inspections | Total Inspections | Sick Colonies | Total Colonies |
| --- | --- | --- | --- | --- |
| Chalkbrood | 179 | 2112 | 114 | 361 |
| Sacbrood | 38 | 2112 | 33 | 361 |
| AFB | 18 | 2112 | 14 | 361 |
| EFB | 7 | 2112 | 6 | 361 |
| DWV | 23 | 2112 | 21 | 361 |
| Other | 37 | 2112 | 36 | 361 |

#### **2.14.1 Categories of disease, Southern Alberta**

| Disease | Disease - Positive Inspections | Total Inspections | Sick Colonies | Total Colonies |
| --- | --- | --- | --- | --- |
| Chalkbrood | 103 | 948 | 59 | 117 |
| Sacbrood | 13 | 948 | 10 | 117 |
| AFB | 0 | 948 | 0 | 117 |
| EFB | 1 | 948 | 1 | 117 |
| DWV | 4 | 948 | 4 | 117 |
| Other | 1 | 948 | 1 | 117 |

#### **2.14.2 Categories of disease, Northern Alberta**

| Disease | Disease - Positive Inspections | Total Inspections | Sick Colonies | Total Colonies |
| --- | --- | --- | --- | --- |
| Chalkbrood | 12 | 842 | 9 | 123 |
| Sacbrood | 1 | 842 | 1 | 123 |
| AFB | 0 | 842 | 0 | 123 |
| EFB | 0 | 842 | 0 | 123 |
| DWV | 2 | 842 | 2 | 123 |
| Other | 5 | 842 | 5 | 123 |

#### **2.14.3 Categories of disease, PEI**

| Disease | Disease - Positive Inspections | Total Inspections | Sick Colonies | Total Colonies |
| --- | --- | --- | --- | --- |
| Chalkbrood | 64 | 322 | 46 | 121 |
| Sacbrood | 24 | 322 | 22 | 121 |
| AFB | 18 | 322 | 14 | 121 |
| EFB | 6 | 322 | 5 | 121 |
| DWV | 17 | 322 | 15 | 121 |
| Other | 31 | 322 | 30 | 121 |

#### **2.15.1 Categories of disease by treatment: untreated control**

| Disease | Disease - Positive Inspections | Total Inspections | Sick Colonies | Total Colonies |
| --- | --- | --- | --- | --- |
| Chalkbrood | 39 | 537 | 29 | 88 |
| Sacbrood | 11 | 537 | 10 | 88 |
| AFB | 3 | 537 | 2 | 88 |
| EFB | 2 | 537 | 2 | 88 |
| DWV | 4 | 537 | 3 | 88 |
| Other | 6 | 537 | 6 | 88 |

#### **2.15.2 Categories of disease by treatment: protein only**

| Disease | Disease - Positive Inspections | Total Inspections | Sick Colonies | Total Colonies |
| --- | --- | --- | --- | --- |
| Chalkbrood | 54 | 466 | 32 | 89 |
| Sacbrood | 5 | 466 | 5 | 89 |
| AFB | 6 | 466 | 5 | 89 |
| EFB | 1 | 466 | 1 | 89 |
| DWV | 9 | 466 | 8 | 89 |
| Other | 9 | 466 | 8 | 89 |

#### **2.15.3 Categories of disease by treatment: fumagillin only**

| Disease | Disease - Positive Inspections | Total Inspections | Sick Colonies | Total Colonies |
| --- | --- | --- | --- | --- |
| Chalkbrood | 48 | 577 | 30 | 89 |
| Sacbrood | 8 | 577 | 7 | 89 |
| AFB | 6 | 577 | 5 | 89 |
| EFB | 3 | 577 | 2 | 89 |
| DWV | 7 | 577 | 7 | 89 |
| Other | 13 | 577 | 13 | 89 |

#### **2.15.4 Categories of disease by treatment: both treatments**

| Disease | Disease - Positive Inspections | Total Inspections | Sick Colonies | Total Colonies |
| --- | --- | --- | --- | --- |
| Chalkbrood | 38 | 532 | 23 | 95 |
| Sacbrood | 14 | 532 | 11 | 95 |
| AFB | 3 | 532 | 2 | 95 |
| EFB | 1 | 532 | 1 | 95 |
| DWV | 3 | 532 | 3 | 95 |
| Other | 9 | 532 | 9 | 95 |

#### **2.16 Number of inspections by category of disease severity**

|  | No sick individuals | <11 sick individuals | 11 - 100 sick individuals | >100 sick individuals |
| --- | --- | --- | --- | --- |
| Chalkbrood | 1935 | 56 | 57 | 66 |
| Sacbrood | 2076 | 23 | 11 | 4 |
| AFB | 2096 | 9 | 2 | 7 |
| EFB | 2107 | 4 | 1 | 2 |
| DWV | 2091 | 20 | 3 | 0 |
| Other’ | 2077 | 34 | 2 | 1 |

# **3. Adult Bee Count Models**

Models that included events and treatements as fixed predictors were
compared to a reference model that included only region and date. The
Design model was the optimized model from Peirson et al, 2024, expressed
as a maximum likelihood model to allow comparisons. Effect estimates
presented in the paper are from the optimized Complete model, which is a
Maximum Likelihood model in section 3.1 and an REML model otherwise.

**Reference Model**

BHPrd<- lme(Adults ~ Region\* Date, random = ~1|Colony.Number,
BHPad, correlation=corAR1(), weights=varIdent(form= ~1|Assessment\*
Region), method = “ML”, control=lmeControl(opt=“optim”))

**Example of a model for a single category of
event**

BHPvarroa<- lme(Adults ~ (Region + Date + AnyVarroa)^2 , random =
~1|Colony.Number, BHPad, correlation=corAR1(), weights=varIdent(form=
~1|Assessment\* Region), method = “ML”,
control=lmeControl(opt=“optim”))

**Complete Model, Before optimization**  
BHPevent<- lme(Adults ~ (Region + Date + Patties + Fumagillin)^4 +
(Region + Date + Patties + Fumagillin)\* (AnyVarroa + Sick +
Queen.Event), random = ~1|Colony.Number, BHPad, correlation=corAR1(),
weights=varIdent(form= ~1|Assessment\* Region), method = “REML”,
control=lmeControl(opt=“optim”))

**Complete Model, after optimization**

BHPevent<- lme(Adults ~ (Region + Patties + Fumagillin)^3 + Date\*
(Region + Fumagillin) + (Date + Region)\* AnyVarroa + Queen.Event + Sick,
random = ~1|Colony.Number, BHPad, correlation=corAR1(),
weights=varIdent(form= ~1|Assessment\* Region), method = “ML”,
control=lmeControl(opt=“optim”))

### **3.1 Maximum Likelihood Model Comparisons**

#### **3.1.1 Varroa model vs reference model**

|  | Model | df | AIC | BIC | logLik | Test | L.Ratio | p-value | R2m | R2c | perc.reduction.unexplained.variability.fxd | perc.reduction.unexplained.variability.total |
| --- | --- | --- | --- | --- | --- | --- | --- | --- | --- | --- | --- | --- |
| BHPrd | 1 | 38 | 27673 | 27872 | -13799 |  | NA | NA | 0.7868 | 0.825 | NA | NA |
| BHPvarroa | 2 | 46 | 27663 | 27904 | -13786 | 1 vs 2 | 26.19 | 0.000975 | 0.7889 | 0.8256 | 0.9589 | 0.3066 |

#### **3.1.2 Disease model vs reference model**

|  | Model | df | AIC | BIC | logLik | Test | L.Ratio | p-value | R2m | R2c | perc.reduction.unexplained.variability.fxd | perc.reduction.unexplained.variability.total |
| --- | --- | --- | --- | --- | --- | --- | --- | --- | --- | --- | --- | --- |
| BHPrd | 1 | 38 | 27673 | 27872 | -13799 |  | NA | NA | 0.7868 | 0.825 | NA | NA |
| BHPsick | 2 | 46 | 27675 | 27916 | -13791 | 1 vs 2 | 14.72 | 0.06488 | 0.7952 | 0.8354 | 3.928 | 5.948 |

#### **3.1.3 Queen model versus reference model**

|  | Model | df | AIC | BIC | logLik | Test | L.Ratio | p-value | R2m | R2c | perc.reduction.unexplained.variability.fxd | perc.reduction.unexplained.variability.total |
| --- | --- | --- | --- | --- | --- | --- | --- | --- | --- | --- | --- | --- |
| BHPrd | 1 | 38 | 27673 | 27872 | -13799 |  | NA | NA | 0.7868 | 0.825 | NA | NA |
| BHPqueen | 2 | 46 | 27671 | 27912 | -13789 | 1 vs 2 | 18.61 | 0.01712 | 0.7895 | 0.8231 | 1.236 | -1.085 |

#### **3.1.4 Design model versus reference model**

|  | Model | df | AIC | BIC | logLik | Test | L.Ratio | p-value | R2m | R2c | perc.reduction.unexplained.variability.fxd | perc.reduction.unexplained.variability.total |
| --- | --- | --- | --- | --- | --- | --- | --- | --- | --- | --- | --- | --- |
| BHPrd | 1 | 38 | 27673 | 27872 | -13799 |  | NA | NA | 0.7868 | 0.825 | NA | NA |
| BHPdesign | 2 | 67 | 27689 | 28040 | -13778 | 1 vs 2 | 41.94 | 0.05682 | 0.7913 | 0.8313 | 2.092 | 3.62 |

#### **3.1.5 Complete Model versus reference model**

|  | Model | df | AIC | BIC | logLik | Test | L.Ratio | p-value | R2m | R2c | perc.reduction.unexplained.variability.fxd | perc.reduction.unexplained.variability.total |
| --- | --- | --- | --- | --- | --- | --- | --- | --- | --- | --- | --- | --- |
| BHPrd | 1 | 38 | 27673 | 27872 | -13799 |  | NA | NA | 0.7868 | 0.825 | NA | NA |
| BHPevent | 2 | 62 | 27656 | 27981 | -13766 | 1 vs 2 | 65.38 | 1.071e-05 | 0.7977 | 0.8351 | 5.117 | 5.743 |

**3.2 Residuals plot of optimized adult bee model**

**3.3 Normal quantile plot of adult bee model**

#### **3.4 Model Effects Summary (adult bee model)**

|  | numDF | denDF | F-value | p-value |
| --- | --- | --- | --- | --- |
| (Intercept) | 1 | 1035 | 441.9 | 0 |
| Region | 2 | 310 | 18.08 | 3.754e-08 |
| Patties | 1 | 310 | 0.06446 | 0.7997 |
| Fumagillin | 1 | 310 | 0.1565 | 0.6927 |
| Date | 5 | 1035 | 109.8 | 0 |
| AnyVarroa | 1 | 1035 | 8.562 | 0.003508 |
| Queen.Event | 1 | 1035 | 7.883 | 0.005085 |
| Sick | 1 | 1035 | 5.969 | 0.01473 |
| Region:Patties | 2 | 310 | 0.3171 | 0.7285 |
| Region:Fumagillin | 2 | 310 | 1.383 | 0.2524 |
| Patties:Fumagillin | 1 | 310 | 0.006312 | 0.9367 |
| Region:Date | 10 | 1035 | 61.28 | 0 |
| Fumagillin:Date | 5 | 1035 | 2.385 | 0.03657 |
| Date:AnyVarroa | 5 | 1035 | 3.529 | 0.003611 |
| Region:AnyVarroa | 2 | 1035 | 3.595 | 0.0278 |
| Region:Patties:Fumagillin | 2 | 310 | 3.861 | 0.02207 |

#### **3.5 Effect estimate contrasts (queen event)**

| contrast | estimate | SE | df | lower.CL | upper.CL | t.ratio | p.value |
| --- | --- | --- | --- | --- | --- | --- | --- |
| Queen.Event1 - Queen.Event0 | -1634 | 581.9 | 1035 | -2776 | -491.9 | -2.808 | 0.005085 |

#### **3.6 Effect estimate contrasts (varroa main effect)**

| contrast | estimate | SE | df | lower.CL | upper.CL | t.ratio | p.value |
| --- | --- | --- | --- | --- | --- | --- | --- |
| Varroa - No Varroa | 47.43 | 575.8 | 1035 | -1083 | 1177 | 0.08237 | 0.9344 |

#### **3.7 Effect estimate contrasts (varroa by region)**

| contrast | Region | estimate | SE | df | lower.CL | upper.CL | t.ratio | p.value |
| --- | --- | --- | --- | --- | --- | --- | --- | --- |
| Varroa - No Varroa | Southern Alberta | 1679 | 1042 | 1035 | -365.7 | 3723 | 1.611 | 0.1074 |
| Varroa - No Varroa | Northern Alberta | 215.3 | 990.4 | 1035 | -1728 | 2159 | 0.2173 | 0.828 |
| Varroa - No Varroa | Prince Edward Island | -1751 | 711.8 | 1035 | -3148 | -354.7 | -2.461 | 0.01404 |

For table 3.7, the significance threshold is 0.05/3 = 0.0167.

#### **3.8 Effect estimate contrasts (varroa by date)**

| contrast | Date | estimate | SE | df | lower.CL | upper.CL | t.ratio | p.value |
| --- | --- | --- | --- | --- | --- | --- | --- | --- |
| Varroa - No Varroa | June 2014 | 2154 | 813.9 | 1035 | 557.4 | 3751 | 2.647 | 0.008242 |
| Varroa - No Varroa | August 2014 | -1005 | 947 | 1035 | -2863 | 853.6 | -1.061 | 0.289 |
| Varroa - No Varroa | May 2015 | -2910 | 1479 | 1035 | -5812 | -8.416 | -1.968 | 0.04934 |
| Varroa - No Varroa | June 2015 | 2292 | 1784 | 1035 | -1209 | 5793 | 1.285 | 0.1992 |
| Varroa - No Varroa | August 2015 | 790.1 | 966.8 | 1035 | -1107 | 2687 | 0.8173 | 0.414 |
| Varroa - No Varroa | May 2016 | -1037 | 1690 | 1035 | -4354 | 2280 | -0.6136 | 0.5396 |

For table 3.8, the significance threshold is 0.05/6 = 0.0083.

#### **3.9 Effect estimate contrasts (visible disease)**

| contrast | estimate | SE | df | lower.CL | upper.CL | t.ratio | p.value |
| --- | --- | --- | --- | --- | --- | --- | --- |
| Sick - Healthy | -856.2 | 350.5 | 1035 | -1544 | -168.5 | -2.443 | 0.01473 |

### **4. Sealed Brood Count Models**

Models that included events and treatements as fixed predictors were
compared to a reference model that included only region and date. The
Design model was the optimized model from Peirson et al, 2024, expressed
as a maximum likelihood model to allow comparisons. Effect estimates
presented in the paper are from the optimized Complete model, which is a
Maximum Likelihood model in section 4.1 and an REML model otherwise.

**Reference Model**

BHPrd<- lme(Worker.Cells ~ Region*Date, random =
~1|Colony.Number, BHPad, correlation=corAR1(), weights=varIdent(form=
~1|Assessment* Region), method = “ML”,
control=lmeControl(opt=“optim”))

**Example of a model for a single category of
event**

BHPvarroa<- lme(Worker.Cells ~ (Region + Date + AnyVarroa)^2,
random = ~1|Colony.Number, BHPad, correlation=corAR1(),
weights=varIdent(form= ~1|Assessment\* Region), method = “ML”,
control=lmeControl(opt=“optim”))

**Model with all events and treatments, after
optimization**

BHPevent<- lme(Worker.Cells ~ (Region + Patties + Fumagillin)^3 +
Date\* (Region + Patties + Fumagillin + Queen.Event) + Region\*
(Queen.Event + AnyVarroa) + Sick, random = ~1|Colony.Number, BHPad,
correlation=corAR1(), weights=varIdent(form= ~1|Assessment\* Region),
method = “ML”, control=lmeControl(opt=“optim”))

### **4.1 Maximum Likelihood Model Comparisons**

#### **4.1.1 Varroa model vs reference model**

|  | Model | df | AIC | BIC | logLik | Test | L.Ratio | p-value | R2m | R2c | perc.reduction.unexplained.variability.fxd | perc.reduction.unexplained.variability.total |
| --- | --- | --- | --- | --- | --- | --- | --- | --- | --- | --- | --- | --- |
| BHPrd | 1 | 38 | 26527 | 26726 | -13226 |  | NA | NA | 0.4854 | 0.5533 | NA | NA |
| BHPvarroa | 2 | 46 | 26521 | 26762 | -13215 | 1 vs 2 | 21.85 | 0.005196 | 0.4897 | 0.5621 | 0.8482 | 1.974 |

#### **4.1.2 Disease model vs reference model**

|  | Model | df | AIC | BIC | logLik | Test | L.Ratio | p-value | R2m | R2c | perc.reduction.unexplained.variability.fxd | perc.reduction.unexplained.variability.total |
| --- | --- | --- | --- | --- | --- | --- | --- | --- | --- | --- | --- | --- |
| BHPrd | 1 | 38 | 26527 | 26726 | -13226 |  | NA | NA | 0.4854 | 0.5533 | NA | NA |
| BHPsick | 2 | 46 | 26532 | 26773 | -13220 | 1 vs 2 | 10.82 | 0.212 | 0.4972 | 0.5677 | 2.301 | 3.239 |

#### **4.1.3 Queen Model vs reference model**

|  | Model | df | AIC | BIC | logLik | Test | L.Ratio | p-value | R2m | R2c | perc.reduction.unexplained.variability.fxd | perc.reduction.unexplained.variability.total |
| --- | --- | --- | --- | --- | --- | --- | --- | --- | --- | --- | --- | --- |
| BHPrd | 1 | 38 | 26527 | 26726 | -13226 |  | NA | NA | 0.4854 | 0.5533 | NA | NA |
| BHPqueen | 2 | 46 | 26413 | 26654 | -13160 | 1 vs 2 | 130.2 | 2.539e-24 | 0.5232 | 0.5696 | 7.35 | 3.665 |

#### **4.1.4 Design model vs reference model**

|  | Model | df | AIC | BIC | logLik | Test | L.Ratio | p-value | R2m | R2c | perc.reduction.unexplained.variability.fxd | perc.reduction.unexplained.variability.total |
| --- | --- | --- | --- | --- | --- | --- | --- | --- | --- | --- | --- | --- |
| BHPrd | 1 | 38 | 26527 | 26726 | -13226 |  | NA | NA | 0.4854 | 0.5533 | NA | NA |
| BHPdesign | 2 | 66 | 26530 | 26875 | -13199 | 1 vs 2 | 53.26 | 0.002737 | 0.5418 | 0.6109 | 10.96 | 12.91 |

#### **4.1.5 Combined Model vs reference model**

|  | Model | df | AIC | BIC | logLik | Test | L.Ratio | p-value | R2m | R2c | perc.reduction.unexplained.variability.fxd | perc.reduction.unexplained.variability.total |
| --- | --- | --- | --- | --- | --- | --- | --- | --- | --- | --- | --- | --- |
| BHPrd | 1 | 38 | 26527 | 26726 | -13226 |  | NA | NA | 0.4854 | 0.5533 | NA | NA |
| BHPevent | 2 | 69 | 26399 | 26759 | -13130 | 1 vs 2 | 190.7 | 6.993e-25 | 0.5849 | 0.6411 | 19.34 | 19.65 |

**4.2 Complete model residuls plot**

**4.3 Complete model normal quantile plot**

#### **4.4 Model effects summary (sealed brood)**

|  | numDF | denDF | F-value | p-value |
| --- | --- | --- | --- | --- |
| (Intercept) | 1 | 1023 | 377.2 | 0 |
| Region | 2 | 310 | 18.97 | 1.683e-08 |
| Patties | 1 | 310 | 12.17 | 0.0005553 |
| Fumagillin | 1 | 310 | 0.001046 | 0.9742 |
| Date | 5 | 1023 | 32.4 | 0 |
| Queen.Event | 1 | 1023 | 6.378 | 0.01171 |
| AnyVarroa | 1 | 1023 | 3.368 | 0.06675 |
| Sick | 1 | 1023 | 4.886 | 0.02729 |
| Region:Patties | 2 | 310 | 2.022 | 0.1341 |
| Region:Fumagillin | 2 | 310 | 1.259 | 0.2853 |
| Patties:Fumagillin | 1 | 310 | 0.0251 | 0.8742 |
| Region:Date | 10 | 1023 | 62.38 | 0 |
| Patties:Date | 5 | 1023 | 4.717 | 0.0002894 |
| Fumagillin:Date | 5 | 1023 | 0.4366 | 0.8232 |
| Date:Queen.Event | 5 | 1023 | 4.341 | 0.0006487 |
| Region:Queen.Event | 2 | 1023 | 12.55 | 4.128e-06 |
| Region:AnyVarroa | 2 | 1023 | 5.239 | 0.005448 |
| Region:Patties:Fumagillin | 2 | 310 | 4.726 | 0.009514 |

#### **4.5 Effect estimate contrasts (queen event, main effect)**

| contrast | estimate | SE | df | lower.CL | upper.CL | t.ratio | p.value |
| --- | --- | --- | --- | --- | --- | --- | --- |
| Queen.Event1 - Queen.Event0 | -2823 | 764.7 | 1023 | -4323 | -1322 | -3.692 | 0.0002345 |

#### **4.6 Effect estimate contrasts (queen event, by date)**

| contrast | Date | estimate | SE | df | lower.CL | upper.CL | t.ratio | p.value |
| --- | --- | --- | --- | --- | --- | --- | --- | --- |
| Queen.Event1 - Queen.Event0 | June 2014 | -4590 | 1238 | 1023 | -7020 | -2161 | -3.708 | 0.0002205 |
| Queen.Event1 - Queen.Event0 | August 2014 | -4773 | 671.8 | 1023 | -6091 | -3455 | -7.104 | 2.263e-12 |
| Queen.Event1 - Queen.Event0 | May 2015 | -3245 | 3519 | 1023 | -10150 | 3661 | -0.922 | 0.3568 |
| Queen.Event1 - Queen.Event0 | June 2015 | -2004 | 844.7 | 1023 | -3661 | -346.4 | -2.372 | 0.01786 |
| Queen.Event1 - Queen.Event0 | August 2015 | -931.8 | 722.2 | 1023 | -2349 | 485.5 | -1.29 | 0.1973 |
| Queen.Event1 - Queen.Event0 | May 2016 | -1394 | 2106 | 1023 | -5527 | 2739 | -0.6619 | 0.5082 |

For table 4.6, the significance threshold is 0.05/6 = 0.0083.

#### **4.7 Effect estimate contrasts (queen event, by region)**

| contrast | Region | estimate | SE | df | lower.CL | upper.CL | t.ratio | p.value |
| --- | --- | --- | --- | --- | --- | --- | --- | --- |
| Queen.Event1 - Queen.Event0 | Southern Alberta | -1323 | 884.5 | 1023 | -3058 | 412.9 | -1.495 | 0.1351 |
| Queen.Event1 - Queen.Event0 | Northern Alberta | -5155 | 813.6 | 1023 | -6751 | -3558 | -6.335 | 3.541e-10 |
| Queen.Event1 - Queen.Event0 | Prince Edward Island | -1991 | 1322 | 1023 | -4585 | 602.5 | -1.507 | 0.1322 |

For table 4.7, the significance threshold is 0.05/3 = 0.0167.

#### **4.8 Effect estimate contrasts (varroa, main effect)**

| contrast | estimate | SE | df | lower.CL | upper.CL | t.ratio | p.value |
| --- | --- | --- | --- | --- | --- | --- | --- |
| Varroa - No Varroa | -858.9 | 313.5 | 1023 | -1474 | -243.7 | -2.739 | 0.006262 |

#### **4.9 Effect estimate contrasts (varroa, by region)**

| contrast | Region | estimate | SE | df | lower.CL | upper.CL | t.ratio | p.value |
| --- | --- | --- | --- | --- | --- | --- | --- | --- |
| Varroa - No Varroa | Southern Alberta | -1062 | 578.5 | 1023 | -2197 | 73.46 | -1.835 | 0.06675 |
| Varroa - No Varroa | Northern Alberta | 396.3 | 454.1 | 1023 | -494.8 | 1287 | 0.8726 | 0.3831 |
| Varroa - No Varroa | Prince Edward Island | -1911 | 585.1 | 1023 | -3059 | -763.2 | -3.267 | 0.001124 |

For table 4.9, the significance threshold is 0.05/3 = 0.0167.

#### **4.10 Effect estimate contrasts (visible disease)**

| contrast | estimate | SE | df | lower.CL | upper.CL | t.ratio | p.value |
| --- | --- | --- | --- | --- | --- | --- | --- |
| Sick - Healthy | -617.5 | 279.3 | 1023 | -1166 | -69.33 | -2.21 | 0.02729 |

### **5. Brood as percent of bee population on the same date**

The model that included all events and treatments as fixed predictors
was compared to a reference model that included only region and date.
Data presented in the paper are from the optimized Complete model, which
is a Maximum Likelihood model in section 5.1 and an REML model
otherwise.

Reference model:

BHPrd<- lme(PercentBrood ~(Date+ Region)^2, random =
~1|Colony.Number, BHPra, correlation=corAR1(), weights=varIdent(form=
~1|Assessment\* Region), method = “ML”,
control=lmeControl(opt=“optim”))

Model with all events and treatments, before optimization:

BHPeventra<- lme(PercentBrood ~ (Region + Date + Patties +
Fumagillin)^4 + (Region + Date + Patties + Fumagillin)\* (AnyVarroa +
Queen.Event+ Sick)), random = ~1|Colony.Number, BHPra,
correlation=corAR1(), weights=varIdent(form= ~1|Assessment\* Region),
method = “ML”, control=lmeControl(opt=“optim”))

Model with all events and treatments, after optimization:

BHPeventra<- lme(PercentBrood ~ (Region + Date + Patties)^3 +
Date\* (Fumagillin + Queen.Event) + Region\* AnyVarroa + Patties\*
Queen.Event + Sick, random = ~1|Colony.Number, BHPra,
correlation=corAR1(), weights=varIdent(form= ~1|Assessment\* Region),
method = “ML”, control=lmeControl(opt=“optim”))

#### **5.1 Maximum likelihood comparison: Complete model vs reference model**

|  | Model | df | AIC | BIC | logLik | Test | L.Ratio | p-value | R2m | R2c | perc.reduction.unexplained.variability.fxd | perc.reduction.unexplained.variability.total |
| --- | --- | --- | --- | --- | --- | --- | --- | --- | --- | --- | --- | --- |
| BHPrd | 1 | 38 | 12456 | 12655 | -6190 |  | NA | NA | 0.6414 | 0.6422 | NA | NA |
| BHPeventra | 2 | 73 | 12400 | 12781 | -6127 | 1 vs 2 | 126.2 | 3.165e-12 | 0.6696 | 0.6727 | 7.883 | 8.498 |

**5.2 Residuals plot of the brood percent model**

**5.3 Normal quantile plot of the brood percent model**

#### **5.4 Model effects summary for sealed brood as a percent of adult bees**

|  | numDF | denDF | F-value | p-value |
| --- | --- | --- | --- | --- |
| (Intercept) | 1 | 1004 | 282.2 | 0 |
| Region | 2 | 315 | 54.46 | 0 |
| Date | 5 | 1004 | 30.2 | 0 |
| Patties | 1 | 315 | 10.73 | 0.001171 |
| Fumagillin | 1 | 315 | 2.186 | 0.1403 |
| Queen.Event | 1 | 1004 | 1.037 | 0.3087 |
| AnyVarroa | 1 | 1004 | 6.916 | 0.008672 |
| Sick | 1 | 1004 | 0.2798 | 0.597 |
| Region:Date | 10 | 1004 | 28.08 | 0 |
| Region:Patties | 2 | 315 | 3.579 | 0.02904 |
| Date:Patties | 5 | 1004 | 3.757 | 0.002246 |
| Date:Fumagillin | 5 | 1004 | 1.204 | 0.3051 |
| Date:Queen.Event | 5 | 1004 | 15.19 | 2.065e-14 |
| Region:AnyVarroa | 2 | 1004 | 3.407 | 0.03353 |
| Patties:Queen.Event | 1 | 1004 | 6.635 | 0.01014 |
| Region:Date:Patties | 10 | 1004 | 2.21 | 0.01536 |

#### **5.5 Effect estimate contrasts (protein treatment, main effect)**

| contrast | estimate | SE | df | lower.CL | upper.CL | t.ratio | p.value |
| --- | --- | --- | --- | --- | --- | --- | --- |
| Yes - No | -5.252 | 2.271 | 315 | -9.72 | -0.7846 | -2.313 | 0.02136 |

#### **5.6 Effect estimate contrasts (protein treatment, by region)**

| contrast | Region | estimate | SE | df | lower.CL | upper.CL | t.ratio | p.value |
| --- | --- | --- | --- | --- | --- | --- | --- | --- |
| Yes - No | Southern Alberta | -3.602 | 2.273 | 315 | -8.075 | 0.8707 | -1.585 | 0.1141 |
| Yes - No | Northern Alberta | -3.532 | 2.362 | 315 | -8.18 | 1.116 | -1.495 | 0.1359 |
| Yes - No | Prince Edward Island | -8.623 | 4.833 | 315 | -18.13 | 0.8858 | -1.784 | 0.07535 |

For table 5.6, the significance threshold is 0.05/3 = 0.0167.

#### **5.7 Effect estimate contrasts (protein treatment, by date)**

| contrast | Date | estimate | SE | df | lower.CL | upper.CL | t.ratio | p.value |
| --- | --- | --- | --- | --- | --- | --- | --- | --- |
| Yes - No | June 2014 | -2.871 | 6.208 | 315 | -15.09 | 9.344 | -0.4625 | 0.6441 |
| Yes - No | August 2014 | -8.607 | 5.271 | 315 | -18.98 | 1.764 | -1.633 | 0.1035 |
| Yes - No | May 2015 | -8.376 | 2.905 | 315 | -14.09 | -2.661 | -2.883 | 0.004206 |
| Yes - No | June 2015 | -3.583 | 3.454 | 315 | -10.38 | 3.213 | -1.037 | 0.3003 |
| Yes - No | August 2015 | -3.958 | 3.128 | 315 | -10.11 | 2.197 | -1.265 | 0.2067 |
| Yes - No | May 2016 | -4.119 | 5.243 | 315 | -14.44 | 6.198 | -0.7855 | 0.4327 |

For table 5.7, the significance threshold is 0.05/6 = 0.0083.

#### **5.8 Effect estimate contrasts (protein treatment by region and date)**

| contrast | Region | Date | estimate | SE | df | lower.CL | upper.CL | t.ratio | p.value |
| --- | --- | --- | --- | --- | --- | --- | --- | --- | --- |
| Yes - No | Southern Alberta | June 2014 | 12.38 | 5.482 | 315 | 1.598 | 23.17 | 2.259 | 0.02457 |
| Yes - No | Northern Alberta | June 2014 | -9.812 | 7.44 | 315 | -24.45 | 4.827 | -1.319 | 0.1882 |
| Yes - No | Prince Edward Island | June 2014 | -11.19 | 15.61 | 315 | -41.9 | 19.53 | -0.7166 | 0.4742 |
| Yes - No | Southern Alberta | August 2014 | -9.815 | 5.927 | 315 | -21.48 | 1.846 | -1.656 | 0.09871 |
| Yes - No | Northern Alberta | August 2014 | -2.015 | 4.15 | 315 | -10.18 | 6.151 | -0.4855 | 0.6277 |
| Yes - No | Prince Edward Island | August 2014 | -13.99 | 13.83 | 315 | -41.2 | 13.21 | -1.012 | 0.3123 |
| Yes - No | Southern Alberta | May 2015 | -5.85 | 3.277 | 315 | -12.3 | 0.5967 | -1.785 | 0.07515 |
| Yes - No | Northern Alberta | May 2015 | -9.221 | 3.811 | 315 | -16.72 | -1.723 | -2.42 | 0.0161 |
| Yes - No | Prince Edward Island | May 2015 | -10.06 | 5.581 | 315 | -21.04 | 0.9224 | -1.802 | 0.07246 |
| Yes - No | Southern Alberta | June 2015 | -10.4 | 2.695 | 315 | -15.7 | -5.098 | -3.859 | 0.0001383 |
| Yes - No | Northern Alberta | June 2015 | 4.038 | 3.401 | 315 | -2.654 | 10.73 | 1.187 | 0.236 |
| Yes - No | Prince Edward Island | June 2015 | -4.387 | 8.778 | 315 | -21.66 | 12.88 | -0.4998 | 0.6176 |
| Yes - No | Southern Alberta | August 2015 | -1.242 | 3.114 | 315 | -7.369 | 4.886 | -0.3987 | 0.6904 |
| Yes - No | Northern Alberta | August 2015 | -3.748 | 3.505 | 315 | -10.64 | 3.148 | -1.069 | 0.2857 |
| Yes - No | Prince Edward Island | August 2015 | -6.883 | 7.932 | 315 | -22.49 | 8.723 | -0.8678 | 0.3862 |
| Yes - No | Southern Alberta | May 2016 | -6.691 | 3.561 | 315 | -13.7 | 0.3167 | -1.879 | 0.06122 |
| Yes - No | Northern Alberta | May 2016 | -0.4362 | 5.271 | 315 | -10.81 | 9.935 | -0.08275 | 0.9341 |
| Yes - No | Prince Edward Island | May 2016 | -5.23 | 13.96 | 315 | -32.69 | 22.23 | -0.3747 | 0.7081 |

For table 5.8, the significance threshold is 0.05/18 =0.0028

#### **5.9 Effect estimate contrasts (protein treatment by queen event)**

| contrast | Queen.Event | estimate | SE | df | lower.CL | upper.CL | t.ratio | p.value |
| --- | --- | --- | --- | --- | --- | --- | --- | --- |
| Yes - No | 0 | -0.5819 | 1.794 | 315 | -4.111 | 2.947 | -0.3244 | 0.7458 |
| Yes - No | 1 | -9.923 | 3.697 | 315 | -17.2 | -2.648 | -2.684 | 0.007664 |

For table 5.9, the significance threshold is 0.05/2 = 0.025.

#### **5.10 Effect estimate contrasts (queen events, main effect)**

| contrast | estimate | SE | df | lower.CL | upper.CL | t.ratio | p.value |
| --- | --- | --- | --- | --- | --- | --- | --- |
| Queen.Event1 - Queen.Event0 | -25.23 | 4.12 | 1004 | -33.31 | -17.14 | -6.123 | 1.314e-09 |

#### **5.11 Effect estimate contrasts (queen events, by date)**

| contrast | Date | estimate | SE | df | lower.CL | upper.CL | t.ratio | p.value |
| --- | --- | --- | --- | --- | --- | --- | --- | --- |
| Queen.Event1 - Queen.Event0 | June 2014 | -17.35 | 12.29 | 1004 | -41.46 | 6.769 | -1.412 | 0.1584 |
| Queen.Event1 - Queen.Event0 | August 2014 | -26.81 | 3.924 | 1004 | -34.51 | -19.11 | -6.832 | 1.454e-11 |
| Queen.Event1 - Queen.Event0 | May 2015 | -62.84 | 15.32 | 1004 | -92.9 | -32.77 | -4.101 | 4.44e-05 |
| Queen.Event1 - Queen.Event0 | June 2015 | 6.481 | 2.661 | 1004 | 1.26 | 11.7 | 2.436 | 0.01502 |
| Queen.Event1 - Queen.Event0 | August 2015 | -0.6523 | 3.39 | 1004 | -7.304 | 6 | -0.1924 | 0.8474 |
| Queen.Event1 - Queen.Event0 | May 2016 | -50.2 | 13.9 | 1004 | -77.48 | -22.93 | -3.612 | 0.0003183 |

For table 5.11, the significance threshold is 0.05/6 = 0.0083

#### **5.12 Effect estimate contrasts (queen events, by protein supplement treatment)**

| contrast | Patties | estimate | SE | df | lower.CL | upper.CL | t.ratio | p.value |
| --- | --- | --- | --- | --- | --- | --- | --- | --- |
| Queen.Event1 - Queen.Event0 | No | -20.56 | 4.408 | 1004 | -29.21 | -11.91 | -4.664 | 3.523e-06 |
| Queen.Event1 - Queen.Event0 | Yes | -29.9 | 4.593 | 1004 | -38.91 | -20.89 | -6.51 | 1.188e-10 |

For table 5.12, the significance threshold is 0.05/2 = 0.025.

#### **5.13 Effect estimate contrasts (varroa, main effect)**

| contrast | estimate | SE | df | lower.CL | upper.CL | t.ratio | p.value |
| --- | --- | --- | --- | --- | --- | --- | --- |
| Varroa - No Varroa | -4.18 | 2.055 | 1004 | -8.212 | -0.1469 | -2.034 | 0.04223 |

#### **5.14 Effect estimate contrasts (varroa, by region)**

| contrast | Region | estimate | SE | df | lower.CL | upper.CL | t.ratio | p.value |
| --- | --- | --- | --- | --- | --- | --- | --- | --- |
| Varroa - No Varroa | Southern Alberta | -8.873 | 3.374 | 1004 | -15.49 | -2.252 | -2.63 | 0.008672 |
| Varroa - No Varroa | Northern Alberta | 1.432 | 2.288 | 1004 | -3.058 | 5.923 | 0.6259 | 0.5315 |
| Varroa - No Varroa | Prince Edward Island | -5.099 | 4.617 | 1004 | -14.16 | 3.961 | -1.104 | 0.2697 |

For table 5.14, the significance threshold is 0.05/3 = 0.167.

### **6. Change in adult bee population**

The model that included all events and treatments as fixed predictors
was compared to a reference model that included only region and date.
Data presented in the paper are from the optimized Complete model, which
is a Maximum Likelihood model in section 6.1 and an REML model
otherwise.

**Note:** Although estimates of R2C and R2M (below at
Table 6.1) suggest that the random variables contribute nothing to
explained variance, comparison of the reference model against the
corresponding models without random effects or correlations showed that
the reference model was the best fit (by AIC, BIC, and likelihood ratio
tests; not shown); hence these variables were retained.

**Reference Model**

BHPrd<- lme(sqrt(PercentAdults) ~ Region\* Date, random =
~1|Colony.Number, BHPgr, correlation=corAR1(), weights=varIdent(form=
~1|Assessment\* Region), method = “ML”,
control=lmeControl(opt=“optim”))

**Complete Model Before Optimization**

BHPeventgr<- lme(sqrt(PercentAdults) ~ (Region + Date + Patties+
Fumagillin)^4 + (Region + Date + Patties+ Fumagillin)\* (preVarroa +
preSick + preQueen), random = ~1|Colony.Number, BHPgr,
correlation=corAR1(), weights=varIdent(form= ~1|Assessment\* Region),
method = “ML”, control=lmeControl(opt=“optim”))

**Optimized Complete Model**

BHPeventgr<- lme(sqrt(PercentAdults) ~ Date\* (Region + Fumagillin
+ preSick) + Patties\* preQueen + preVarroa , random = ~1|Colony.Number,
BHPgr, correlation=corAR1(), weights=varIdent(form= ~1|Assessment\*
Region), method = “ML”, control=lmeControl(opt=“optim”))

#### **6.1 Maximum likelihood comparison: Complete model versus reference model**

|  | Model | df | AIC | BIC | logLik | Test | L.Ratio | p-value | R2m | R2c | perc.reduction.unexplained.variability.fxd | perc.reduction.unexplained.variability.total |
| --- | --- | --- | --- | --- | --- | --- | --- | --- | --- | --- | --- | --- |
| BHPrd | 1 | 32 | 4829 | 4989 | -2383 |  | NA | NA | 0.6077 | 0.6077 | NA | NA |
| BHPeventgr | 2 | 46 | 4818 | 5047 | -2363 | 1 vs 2 | 39.51 | 0.0003043 | 0.6361 | 0.6361 | 7.23 | 7.23 |

**6.2 Residuals plot of the adult percent model**

**6.3 Normal quantile plot of the adult percent model**

#### **6.4 Model effects summary for adult bees as a percent of adult bee count on the previous date**

|  | numDF | denDF | F-value | p-value |
| --- | --- | --- | --- | --- |
| (Intercept) | 1 | 745 | 1320 | 0 |
| Date | 4 | 745 | 47.21 | 0 |
| Region | 2 | 294 | 40.03 | 4.441e-16 |
| Fumagillin | 1 | 294 | 4.032 | 0.04555 |
| preSick | 1 | 745 | 4.131 | 0.04247 |
| Patties | 1 | 294 | 0.6616 | 0.4166 |
| preQueen | 1 | 745 | 5.264 | 0.02205 |
| preVarroa | 1 | 745 | 5.127 | 0.02385 |
| Date:Region | 8 | 745 | 81.16 | 0 |
| Date:Fumagillin | 4 | 745 | 1.321 | 0.2605 |
| Date:preSick | 4 | 745 | 3.589 | 0.006574 |
| Patties:preQueen | 1 | 745 | 8.349 | 0.003971 |

#### **6.5 Effect estimate contrasts (protein supplement main effect)**

| contrast | estimate | SE | df | lower.CL | upper.CL | t.ratio | p.value |
| --- | --- | --- | --- | --- | --- | --- | --- |
| Yes - No | -13.56 | 3.854 | 294 | -21.14 | -5.974 | -3.518 | 0.0005034 |

#### **6.6 Effect estimate supplement (protein supplement by queen event)**

| contrast | preQueen | estimate | SE | df | lower.CL | upper.CL | t.ratio | p.value |
| --- | --- | --- | --- | --- | --- | --- | --- | --- |
| Yes - No | 0 | -1.786 | 2.196 | 294 | -6.108 | 2.535 | -0.8136 | 0.4166 |
| Yes - No | 1 | -25.33 | 7.639 | 294 | -40.37 | -10.3 | -3.316 | 0.001027 |

For table 6.6, the significance threshold is 0.05/2 = 0.025.

#### **6.7 Effect estimate contrasts (fumagillin main effect)**

| contrast | estimate | SE | df | lower.CL | upper.CL | t.ratio | p.value |
| --- | --- | --- | --- | --- | --- | --- | --- |
| Yes - No | 1.367 | 2.073 | 294 | -2.712 | 5.446 | 0.6596 | 0.51 |

#### **6.8 Effect estimate contrasts (fumagillin by date)**

| contrast | Date | estimate | SE | df | lower.CL | upper.CL | t.ratio | p.value |
| --- | --- | --- | --- | --- | --- | --- | --- | --- |
| Yes - No | May 2015 | 2.145 | 4.922 | 294 | -7.541 | 11.83 | 0.4359 | 0.6632 |
| Yes - No | June 2015 | 2.437 | 5.521 | 294 | -8.429 | 13.3 | 0.4414 | 0.6592 |
| Yes - No | August 2015 | -4.425 | 6.229 | 294 | -16.68 | 7.835 | -0.7103 | 0.4781 |
| Yes - No | May 2016 | 5.31 | 3.174 | 294 | -0.9354 | 11.56 | 1.673 | 0.09533 |

For table 6.8, the significance threshold is 0.05/4 = 0.0125. Note
that the estimates for fumagillin do not include dates prior to the
first fumagillin application in fall 2014.

#### **6.9 Effect estimate contrasts (disease main effect)**

| contrast | estimate | SE | df | lower.CL | upper.CL | t.ratio | p.value |
| --- | --- | --- | --- | --- | --- | --- | --- |
| Sick - Healthy | 2.941 | 3.969 | 294 | -4.87 | 10.75 | 0.7411 | 0.4593 |

#### **6.10 Effect estimate contrasts (disease by date)**

| contrast | Date | estimate | SE | df | lower.CL | upper.CL | t.ratio | p.value |
| --- | --- | --- | --- | --- | --- | --- | --- | --- |
| Sick - Healthy | August 2014 | 22.3 | 11.12 | 294 | 0.4156 | 44.19 | 2.005 | 0.04583 |
| Sick - Healthy | May 2015 | -6.433 | 7.791 | 294 | -21.77 | 8.899 | -0.8258 | 0.4096 |
| Sick - Healthy | June 2015 | -13.35 | 8.737 | 294 | -30.54 | 3.844 | -1.528 | 0.1276 |
| Sick - Healthy | August 2015 | 24.36 | 10.48 | 294 | 3.733 | 44.98 | 2.324 | 0.02079 |
| Sick - Healthy | May 2016 | -12.17 | 7.651 | 294 | -27.23 | 2.889 | -1.59 | 0.1128 |

For table 6.10, the significance threshold is 0.05/5 = 0.01.

#### **6.11 Effect estimate contrasts (varroa main effect)**

| contrast | estimate | SE | df | lower.CL | upper.CL | t.ratio | p.value |
| --- | --- | --- | --- | --- | --- | --- | --- |
| Varroa - No Varroa | -10.39 | 4.538 | 294 | -19.32 | -1.459 | -2.29 | 0.02275 |

#### **6.12 Effect estimate contrasts (supersedure main effect)**

| contrast | estimate | SE | df | lower.CL | upper.CL | t.ratio | p.value |
| --- | --- | --- | --- | --- | --- | --- | --- |
| preQueen1 - preQueen0 | 2.615 | 4.398 | 294 | -6.041 | 11.27 | 0.5945 | 0.5526 |

#### **6.13 Effect estimate contrasts (supersedure by protein supplement treatment)**

| contrast | Patties | estimate | SE | df | lower.CL | upper.CL | t.ratio | p.value |
| --- | --- | --- | --- | --- | --- | --- | --- | --- |
| preQueen1 - preQueen0 | No | 14.39 | 6.433 | 294 | 1.727 | 27.05 | 2.237 | 0.02606 |
| preQueen1 - preQueen0 | Yes | -9.158 | 5.547 | 294 | -20.07 | 1.759 | -1.651 | 0.09981 |

For table 6.13, the significance threshold is 0.05/2 = 0.025.

### **7. Honey Production**

For honey production, event variables with “pre” as a prefix refer to
events detected in June (that is, the event occurred before the honey
flow), while event variables without “pre” refer to detections in August
(that is, the event was detected at the end of the honey flow).

The design model is the model of honey production presented in
Peirson et al, 2024, here treated as a maximum likelihood model to allow
model comparisons

**Reference Model:**

RDBee <- lme(Honey ~ (Region + Date)^2, Xhp, random = ~ 1 |
Colony.Number, weights = varIdent(form = ~ 1 | Region \* Date), method =
“ML”)

**Example of an “event” model:**

QueenBee <- lme(Honey ~ (Region + Date + Queen.Event)^3, Xhp,
random = ~ 1 | Colony.Number, weights = varIdent(form = ~ 1 | Region \*
Date), method = “ML”)

**Model with all events and treatments, before
optimization:**

completemodel <- lme(Honey ~ (Region + Date + Patties +
Fumagillin)^4 + (Region + Date + Patties + Fumagillin)\* (Queen.Event +
preQueen + AnyVarroa + preVarroa + Sick + preSick), Xhp, random = ~ 1 |
Colony.Number, method = “ML”, weights = varIdent(form = ~ 1 | Region \*
Date))

**Model with all events and treatments, after
optimization**

completemodel <- lme(Honey ~ (Region + Date) \* Patties \*
Fumagillin + Region \* Date + Region\* (Queen.Event + preQueen) + Date\*
AnyVarroa + preVarroa + Sick + preSick, Xhp, random = ~ 1 |
Colony.Number, method = “ML”, weights = varIdent(form = ~ 1 | Region \*
Date))

- Optimization means the sequential removal of non-significant fixed
  effects, beginning with the highest order interactions, until only
  effects estimated as statistically signficant (and main effects) remain.
  The interaction of fumagillin with date was retained even if
  non-significant because the initial fumagillin treatment occurred after
  the first summer of data collection.

### **7.1 Maximum likelihood model comparisons**

#### **7.1.1 preQueen model vs reference model**

|  | Model | df | AIC | BIC | logLik | Test | L.Ratio | p-value | R2m | R2c | perc.reduction.unexplained.variability.fxd | perc.reduction.unexplained.variability.total |
| --- | --- | --- | --- | --- | --- | --- | --- | --- | --- | --- | --- | --- |
| RDBee | 1 | 9 | 3534 | 3569 | -1758 |  | NA | NA | 0.8539 | 0.9314 | NA | NA |
| preQueenBee | 2 | 13 | 3525 | 3576 | -1749 | 1 vs 2 | 17.25 | 0.001726 | 0.8596 | 0.9347 | 3.915 | 4.875 |

#### **7.1.2 Queen event model vs reference model**

|  | Model | df | AIC | BIC | logLik | Test | L.Ratio | p-value | R2m | R2c | perc.reduction.unexplained.variability.fxd | perc.reduction.unexplained.variability.total |
| --- | --- | --- | --- | --- | --- | --- | --- | --- | --- | --- | --- | --- |
| RDBee | 1 | 9 | 3534 | 3569 | -1758 |  | NA | NA | 0.8539 | 0.9314 | NA | NA |
| QueenBee | 2 | 13 | 3531 | 3582 | -1753 | 1 vs 2 | 10.47 | 0.03323 | 0.8636 | 0.9433 | 6.629 | 17.29 |

#### **7.1.3 preSick model vs reference model**

|  | Model | df | AIC | BIC | logLik | Test | L.Ratio | p-value | R2m | R2c | perc.reduction.unexplained.variability.fxd | perc.reduction.unexplained.variability.total |
| --- | --- | --- | --- | --- | --- | --- | --- | --- | --- | --- | --- | --- |
| RDBee | 1 | 9 | 3534 | 3569 | -1758 |  | NA | NA | 0.8539 | 0.9314 | NA | NA |
| preSickBee | 2 | 13 | 3524 | 3575 | -1749 | 1 vs 2 | 17.65 | 0.001447 | 0.8692 | 0.9423 | 10.47 | 15.96 |

#### **7.1.4 Sick model vs reference model**

|  | Model | df | AIC | BIC | logLik | Test | L.Ratio | p-value | R2m | R2c | perc.reduction.unexplained.variability.fxd | perc.reduction.unexplained.variability.total |
| --- | --- | --- | --- | --- | --- | --- | --- | --- | --- | --- | --- | --- |
| RDBee | 1 | 9 | 3534 | 3569 | -1758 |  | NA | NA | 0.8539 | 0.9314 | NA | NA |
| SickBee | 2 | 13 | 3528 | 3579 | -1751 | 1 vs 2 | 14.06 | 0.007098 | 0.8685 | 0.9342 | 10 | 4.109 |

#### **7.1.5 preMite model vs reference model**

|  | Model | df | AIC | BIC | logLik | Test | L.Ratio | p-value | R2m | R2c | perc.reduction.unexplained.variability.fxd | perc.reduction.unexplained.variability.total |
| --- | --- | --- | --- | --- | --- | --- | --- | --- | --- | --- | --- | --- |
| RDBee | 1 | 9 | 3534 | 3569 | -1758 |  | NA | NA | 0.8539 | 0.9314 | NA | NA |
| preMiteBee | 2 | 13 | 3539 | 3591 | -1757 | 1 vs 2 | 2.345 | 0.6725 | 0.8546 | 0.9298 | 0.4828 | -2.275 |

#### **7.1.6 Varroa model vs reference model**

|  | Model | df | AIC | BIC | logLik | Test | L.Ratio | p-value | R2m | R2c | perc.reduction.unexplained.variability.fxd | perc.reduction.unexplained.variability.total |
| --- | --- | --- | --- | --- | --- | --- | --- | --- | --- | --- | --- | --- |
| RDBee | 1 | 9 | 3534 | 3569 | -1758 |  | NA | NA | 0.8539 | 0.9314 | NA | NA |
| MiteBee | 2 | 13 | 3529 | 3580 | -1752 | 1 vs 2 | 12.62 | 0.01329 | 0.8558 | 0.9387 | 1.271 | 10.62 |

#### **7.1.7 Design model vs reference model**

|  | Model | df | AIC | BIC | logLik | Test | L.Ratio | p-value | R2m | R2c | perc.reduction.unexplained.variability.fxd | perc.reduction.unexplained.variability.total |
| --- | --- | --- | --- | --- | --- | --- | --- | --- | --- | --- | --- | --- |
| RDBee | 1 | 9 | 3534 | 3569 | -1758 |  | NA | NA | 0.8539 | 0.9314 | NA | NA |
| paper1model | 2 | 18 | 3538 | 3609 | -1751 | 1 vs 2 | 13.57 | 0.1383 | 0.8591 | 0.9335 | 3.529 | 3.069 |

#### **7.1.8 Complete model vs reference model**

|  | Model | df | AIC | BIC | logLik | Test | L.Ratio | p-value | R2m | R2c | perc.reduction.unexplained.variability.fxd | perc.reduction.unexplained.variability.total |
| --- | --- | --- | --- | --- | --- | --- | --- | --- | --- | --- | --- | --- |
| RDBee | 1 | 9 | 3534 | 3569 | -1758 |  | NA | NA | 0.8539 | 0.9314 | NA | NA |
| completemodel | 2 | 27 | 3491 | 3597 | -1719 | 1 vs 2 | 78.73 | 1.429e-09 | 0.8951 | 0.9684 | 28.2 | 53.98 |

**7.2 Residuals plot of the hony model**

**7.3 Normal quantile plot of the honey model**

#### **7.4 Model effects summary for honey production**

|  | numDF | denDF | F-value | p-value |
| --- | --- | --- | --- | --- |
| (Intercept) | 1 | 214 | 101.8 | 0 |
| Region | 1 | 214 | 142.1 | 0 |
| Date | 1 | 141 | 14.94 | 0.000169 |
| Patties | 1 | 214 | 0.4744 | 0.4917 |
| Fumagillin | 1 | 214 | 0.03694 | 0.8478 |
| Queen.Event | 1 | 141 | 6.339 | 0.01293 |
| preQueen | 1 | 141 | 0.2961 | 0.5872 |
| AnyVarroa | 1 | 141 | 1.547 | 0.2156 |
| preVarroa | 1 | 141 | 0.9626 | 0.3282 |
| Sick | 1 | 141 | 9.264 | 0.002789 |
| preSick | 1 | 141 | 13.12 | 0.0004063 |
| Region:Patties | 1 | 214 | 4.8 | 0.02955 |
| Date:Patties | 1 | 141 | 2.44 | 0.1206 |
| Region:Fumagillin | 1 | 214 | 5.428 | 0.02075 |
| Date:Fumagillin | 1 | 141 | 0.6163 | 0.4338 |
| Patties:Fumagillin | 1 | 214 | 0.2191 | 0.6402 |
| Region:Date | 1 | 141 | 118.6 | 0 |
| Region:Queen.Event | 1 | 141 | 4.554 | 0.03457 |
| Region:preQueen | 1 | 141 | 12.97 | 0.0004367 |
| Date:AnyVarroa | 1 | 141 | 4.624 | 0.03324 |
| Region:Patties:Fumagillin | 1 | 214 | 7.832 | 0.005602 |
| Date:Patties:Fumagillin | 1 | 141 | 0.0005582 | 0.9812 |

#### **7.5 Effect estimate contrasts (supersedure during the honey flow main effect)**

| contrast | estimate | SE | df | lower.CL | upper.CL | t.ratio | p.value |
| --- | --- | --- | --- | --- | --- | --- | --- |
| Queen.Event1 - Queen.Event0 | 3.465 | 3.745 | 141 | -3.939 | 10.87 | 0.9251 | 0.3565 |

#### **7.6 Effect estimate contrasts (supersedure during the honey flow, by region)**

| contrast | Region | estimate | SE | df | lower.CL | upper.CL | t.ratio | p.value |
| --- | --- | --- | --- | --- | --- | --- | --- | --- |
| Queen.Event1 - Queen.Event0 | Southern Alberta | 11.37 | 4.515 | 141 | 2.441 | 20.29 | 2.518 | 0.01293 |
| Queen.Event1 - Queen.Event0 | Northern Alberta | -4.438 | 5.924 | 141 | -16.15 | 7.273 | -0.7492 | 0.455 |

For table 7.6, the significance threshold is 0.05/2 = 0.025.

#### **7.7 Effect estimate contrasts (supersedure before the honey flow, main effect)**

| contrast | estimate | SE | df | lower.CL | upper.CL | t.ratio | p.value |
| --- | --- | --- | --- | --- | --- | --- | --- |
| preQueen1 - preQueen0 | -26.66 | 6.712 | 141 | -39.93 | -13.39 | -3.972 | 0.000113 |

#### **7.8 Effect estimate contrasts (supersedure before the honey flow, by region)**

| contrast | Region | estimate | SE | df | lower.CL | upper.CL | t.ratio | p.value |
| --- | --- | --- | --- | --- | --- | --- | --- | --- |
| preQueen1 - preQueen0 | Southern Alberta | -2.471 | 4.542 | 141 | -11.45 | 6.507 | -0.5441 | 0.5872 |
| preQueen1 - preQueen0 | Northern Alberta | -50.86 | 12.64 | 141 | -75.84 | -25.87 | -4.024 | 9.285e-05 |

For table 7.8, the significance threshold is 0.05/2 = 0.025.

#### **7.9 Effect estimate contrasts (varroa detected in August, main effect)**

| contrast | estimate | SE | df | lower.CL | upper.CL | t.ratio | p.value |
| --- | --- | --- | --- | --- | --- | --- | --- |
| Varroa - No Varroa | 13.33 | 3.647 | 141 | 6.116 | 20.53 | 3.654 | 0.0003633 |

#### **7.10 Effect estimate contrasts (varroa detected in August, by year)**

| contrast | Date | estimate | SE | df | lower.CL | upper.CL | t.ratio | p.value |
| --- | --- | --- | --- | --- | --- | --- | --- | --- |
| Varroa - No Varroa | August 2014 | 5.337 | 4.291 | 141 | -3.145 | 13.82 | 1.244 | 0.2156 |
| Varroa - No Varroa | August 2015 | 21.31 | 5.983 | 141 | 9.486 | 33.14 | 3.563 | 0.0005015 |

For table 7.10, the significance threshold is 0.05/2 = 0.025.

#### **7.11 Effect estimate contrasts (varroa detected in June, main effect)**

| contrast | estimate | SE | df | lower.CL | upper.CL | t.ratio | p.value |
| --- | --- | --- | --- | --- | --- | --- | --- |
| Varroa - No Varroa | -6.079 | 6.196 | 141 | -18.33 | 6.171 | -0.9811 | 0.3282 |

#### **7.12 Effect estimate contrasts (visible disease detected after the honey flow, main effect)**

| contrast | estimate | SE | df | lower.CL | upper.CL | t.ratio | p.value |
| --- | --- | --- | --- | --- | --- | --- | --- |
| Sick - Healthy | -11.89 | 3.907 | 141 | -19.62 | -4.168 | -3.044 | 0.002789 |

#### **7.13 Effect estimage contrasts (visible disease detected before the honey flow, main effect)**

| contrast | estimate | SE | df | lower.CL | upper.CL | t.ratio | p.value |
| --- | --- | --- | --- | --- | --- | --- | --- |
| Sick - Healthy | -9.628 | 2.658 | 141 | -14.88 | -4.374 | -3.622 | 0.0004063 |

#### **7.14 Effect estimate contrasts (fumagillin in 2015)**

| contrast | Region | Date | Patties | estimate | SE | df | lower.CL | upper.CL | t.ratio | p.value |
| --- | --- | --- | --- | --- | --- | --- | --- | --- | --- | --- |
| Yes - No | Southern Alberta | August 2015 | No | 4.143 | 6.595 | 141 | -8.896 | 17.18 | 0.6281 | 0.5309 |
| Yes - No | Northern Alberta | August 2015 | No | 23.54 | 8.331 | 141 | 7.07 | 40.01 | 2.826 | 0.005406 |
| Yes - No | Southern Alberta | August 2015 | Yes | 1.956 | 6.733 | 141 | -11.35 | 15.27 | 0.2905 | 0.7719 |
| Yes - No | Northern Alberta | August 2015 | Yes | -12.83 | 9.533 | 141 | -31.68 | 6.014 | -1.346 | 0.1805 |

### **8. Survival Models**

There is no reference model based on region and date alone for the
survival model because in the time to event analysis, date is the
dependent variable, and because region is a stratified factor
(stratified factors are used to estimate different baseline hazards, but
there is no estimate for their effect; in essence the model with
strata(Region) as a factor is the null model)

**Design Model**

The design model is the model from Peirson et al, 2024, except that
there was no cluster factor in this case. The cluster factor
(“ColonyGroup”) was included in the previous analysis to account for
split colonies; risks of death for daughter and parent colonies were
assumed not to be independent. In the present analysis, daughter
colonies are excluded because observations of visible disease and varroa
were not routinely recorded on these colonies. Consequently, no
clustering is required.

DesignModel <- coxph(Surv(tstart, tstop, death) ~ strata(Region) \*
Patties + Fume, newBHPsurv)

**Example of a single factor model**

QueenModel<- coxph(Surv(tstart, tstop, death) ~ strata(Region) +
queen, newBHPsurv)

The queen and mite models did not converge when interactions between
region and the time dependent covariate were included, so interactions
were dropped for those models

**Complete model before simplification**

cphfr<- coxph(Surv(tstart, tstop, death) ~
(strata(Region)+Patties+Fume)^3 + (strata(Region) + Patties +
Fume)\*(sick + mites + queen), newBHPsurv)

The complete model without simplification could not be relied on
because it produced convergence warnings. Interactions between model
terms were removed until a model that converged was identified;
subsequently non-significant interaction terms were successively removed
until the model contained only main effects and significant
interactions.

**Complete model after simplification**

cphfr<- coxph(Surv(tstart, tstop, death) ~ strata(Region)\*(Patties
+ sick) + Fume + mites + queen, newBHPsurv)

#### **8.1 Comparison of Survival Models**

|  | loglik | AIC | BIC | Cox R2 |
| --- | --- | --- | --- | --- |
| Coxqueen | -650.5 | 1303 | 1306 | 0.01687 |
| Coxmites | -650.8 | 1304 | 1307 | 0.01385 |
| Coxsick | -646.1 | 1298 | 1307 | 0.07036 |
| CoxFume | -651.4 | 1309 | 1318 | 0.006442 |
| CoxPatties | -646.3 | 1299 | 1308 | 0.06835 |
| Coxdesign | -646.2 | 1300 | 1313 | 0.06927 |
| CoxComplete | -638.2 | 1294 | 1322 | 0.1603 |

#### **8.2 Model effects summary for colony survival**

Analysis of Deviance Table (Type II tests)


|  | Df | Chisq | Pr(>Chisq) |
| --- | --- | --- | --- |
| Patties | 1 | 3.59 | 0.05812 |
| sick | 1 | 5.324 | 0.02104 |
| Fume | 1 | 0.1589 | 0.6901 |
| mites | 1 | 2.264 | 0.1324 |
| queen | 1 | 2.214 | 0.1368 |
| strata(Region):Patties | 2 | 7.191 | 0.02745 |
| strata(Region):sick | 2 | 7.623 | 0.02211 |

#### **8.3 Test of proportional hazards assumption**

|  | chisq | df | p |
| --- | --- | --- | --- |
| Patties | 2.9 | 1 | 0.08859 |
| sick | 1.57 | 1 | 0.2102 |
| Fume | 0.206 | 1 | 0.6499 |
| mites | 0.7174 | 1 | 0.397 |
| queen | 0.04211 | 1 | 0.8374 |
| strata(Region):Patties | 7.043 | 2 | 0.02956 |
| strata(Region):sick | 4.812 | 2 | 0.09018 |
| GLOBAL | 13.31 | 9 | 0.1489 |

#### **8.4 Survival model estimates**

Fitting Proportional Hazards Regression Model: Surv(tstart,
tstop, death) ~ strata(Region) \* (Patties + sick) + Fume + mites +
queen


|  | coef | exp(coef) | se(coef) | z | p |
| --- | --- | --- | --- | --- | --- |
| PattiesYes | 0.06298 | 1.065 | 0.3548 | 0.1775 | 0.8591 |
| sickSick | 1.739 | 5.693 | 0.4952 | 3.512 | 0.0004445 |
| Fume | -0.07104 | 0.9314 | 0.1782 | -0.3987 | 0.6901 |
| mitesVarroa | 0.3598 | 1.433 | 0.2391 | 1.505 | 0.1324 |
| queen | -0.4395 | 0.6444 | 0.2954 | -1.488 | 0.1368 |
| strata(Region)Northern Alberta:PattiesYes | 0.8354 | 2.306 | 0.4485 | 1.863 | 0.06252 |
| strata(Region)Prince Edward Island:PattiesYes | -0.1101 | 0.8957 | 0.4318 | -0.255 | 0.7988 |
| strata(Region)Northern Alberta:sickSick | -1.481 | 0.2275 | 1.14 | -1.299 | 0.1939 |
| strata(Region)Prince Edward Island:sickSick | -1.544 | 0.2136 | 0.5617 | -2.748 | 0.005987 |

Likelihood ratio test=27.43 on 9 df, p=0.001185149 n= 2122, number of
events= 157 (316 observations deleted due to missingness)

#### **8.5 Hazard ratio for visible disease (main effect)**

| Disease Effect | Region | ratio | SE | df | asymp.LCL | asymp.UCL | null | z.ratio | p.value |
| --- | --- | --- | --- | --- | --- | --- | --- | --- | --- |
| Sick / Healthy | Southern Alberta | 5.693 | 2.819 | Inf | 2.157 | 15.03 | 1 | 3.512 | 0.0004445 |
| Sick / Healthy | Northern Alberta | 1.295 | 1.329 | Inf | 0.1733 | 9.679 | 1 | 0.252 | 0.8011 |
| Sick / Healthy | Prince Edward Island | 1.216 | 0.322 | Inf | 0.7235 | 2.043 | 1 | 0.7381 | 0.4605 |

#### **8.6 Hazard ratio for visible disease (by region)**

| Disease Effect | Region | ratio | SE | df | asymp.LCL | asymp.UCL | null | z.ratio | p.value |
| --- | --- | --- | --- | --- | --- | --- | --- | --- | --- |
| Sick / Healthy | Southern Alberta | 5.693 | 2.819 | Inf | 2.157 | 15.03 | 1 | 3.512 | 0.0004445 |
| Sick / Healthy | Northern Alberta | 1.295 | 1.329 | Inf | 0.1733 | 9.679 | 1 | 0.252 | 0.8011 |
| Sick / Healthy | Prince Edward Island | 1.216 | 0.322 | Inf | 0.7235 | 2.043 | 1 | 0.7381 | 0.4605 |

Significance threshold for Table 8.6 is 0.05/3 = 0.0167.

#### **8.7 Hazard ratio for protein supplements (main effect)**

| Protein Effect | ratio | SE | df | asymp.LCL | asymp.UCL | null | z.ratio | p.value |
| --- | --- | --- | --- | --- | --- | --- | --- | --- |
| Yes / No | 1.356 | 0.2311 | Inf | 0.9712 | 1.894 | 1 | 1.788 | 0.07371 |

#### **8.8 Hazard ratio for protein supplements (by region)**

| Protein Effect | Region | ratio | SE | df | asymp.LCL | asymp.UCL | null | z.ratio | p.value |
| --- | --- | --- | --- | --- | --- | --- | --- | --- | --- |
| Yes / No | Southern Alberta | 1.065 | 0.3778 | Inf | 0.5313 | 2.135 | 1 | 0.1775 | 0.8591 |
| Yes / No | Northern Alberta | 2.456 | 0.674 | Inf | 1.434 | 4.205 | 1 | 3.273 | 0.001063 |
| Yes / No | Prince Edward Island | 0.954 | 0.2345 | Inf | 0.5893 | 1.544 | 1 | -0.1917 | 0.848 |

#### **8.9 Hazard ratio for fumagillin (main effect)**

| Fumagillin Effect | ratio | SE | df | asymp.LCL | asymp.UCL | null | z.ratio | p.value |
| --- | --- | --- | --- | --- | --- | --- | --- | --- |
| Fume1 / Fume0 | 0.9314 | 0.166 | Inf | 0.6568 | 1.321 | 1 | -0.3987 | 0.6901 |

#### **8.10 Hazard ratio varroa detection (main effect)**

| Varroa Effect | ratio | SE | df | asymp.LCL | asymp.UCL | null | z.ratio | p.value |
| --- | --- | --- | --- | --- | --- | --- | --- | --- |
| Varroa / No Varroa | 1.433 | 0.3427 | Inf | 0.8969 | 2.29 | 1 | 1.505 | 0.1324 |

#### **8.11 Hazard ratio for supersedure (main effect)**

| Queen Effect | ratio | SE | df | asymp.LCL | asymp.UCL | null | z.ratio | p.value |
| --- | --- | --- | --- | --- | --- | --- | --- | --- |
| queen1 / queen0 | 0.6444 | 0.1903 | Inf | 0.3612 | 1.15 | 1 | -1.488 | 0.1368 |

**Session Info**

```
## R version 4.4.1 (2024-06-14)
## Platform: x86_64-pc-linux-gnu
## Running under: Ubuntu 22.04.3 LTS
## 
## Matrix products: default
## BLAS:   /usr/lib/x86_64-linux-gnu/blas/libblas.so.3.10.0 
## LAPACK: /usr/lib/x86_64-linux-gnu/lapack/liblapack.so.3.10.0
## 
## locale:
##  [1] LC_CTYPE=en_CA.UTF-8       LC_NUMERIC=C              
##  [3] LC_TIME=en_CA.UTF-8        LC_COLLATE=en_CA.UTF-8    
##  [5] LC_MONETARY=en_CA.UTF-8    LC_MESSAGES=en_CA.UTF-8   
##  [7] LC_PAPER=en_CA.UTF-8       LC_NAME=C                 
##  [9] LC_ADDRESS=C               LC_TELEPHONE=C            
## [11] LC_MEASUREMENT=en_CA.UTF-8 LC_IDENTIFICATION=C       
## 
## time zone: America/Winnipeg
## tzcode source: system (glibc)
## 
## attached base packages:
## [1] stats     graphics  grDevices utils     datasets  methods   base     
## 
## other attached packages:
## [1] car_3.1-3      carData_3.0-5  CoxR2_1.0      MuMIn_1.48.4   survival_3.7-0
## [6] emmeans_1.10.5 nlme_3.1-166   pander_0.6.5   dplyr_1.1.4   
## 
## loaded via a namespace (and not attached):
##  [1] Matrix_1.6-5       jsonlite_1.8.8     highr_0.11         compiler_4.4.1    
##  [5] tidyselect_1.2.0   Rcpp_1.0.12        jquerylib_0.1.4    splines_4.4.1     
##  [9] yaml_2.3.8         fastmap_1.1.1      lattice_0.22-6     R6_2.5.1          
## [13] generics_0.1.3     Formula_1.2-5      knitr_1.45         tibble_3.2.1      
## [17] bslib_0.6.1        pillar_1.9.0       rlang_1.1.3        utf8_1.2.4        
## [21] cachem_1.0.8       xfun_0.41          sass_0.4.8         estimability_1.4.1
## [25] cli_3.6.2          magrittr_2.0.3     digest_0.6.34      grid_4.4.1        
## [29] rstudioapi_0.15.0  mvtnorm_1.2-4      xtable_1.8-4       lifecycle_1.0.4   
## [33] vctrs_0.6.5        evaluate_0.23      glue_1.7.0         abind_1.4-8       
## [37] stats4_4.4.1       fansi_1.0.6        rmarkdown_2.25     tools_4.4.1       
## [41] pkgconfig_2.0.3    htmltools_0.5.7
```
