# Supplementary figures and images for: Supersedure, mites, and visible disease in Apis mellifera (Hymenoptera: Apidae) colonies explain differences in productivity and survival, but the effects may be difficult to see
Source: J Econ Entomol. 2025 Jun 13;118(4):1463–74. doi: 10.1093/jee/toaf094 (PMC12397970; doi:10.1093/jee/toaf094)

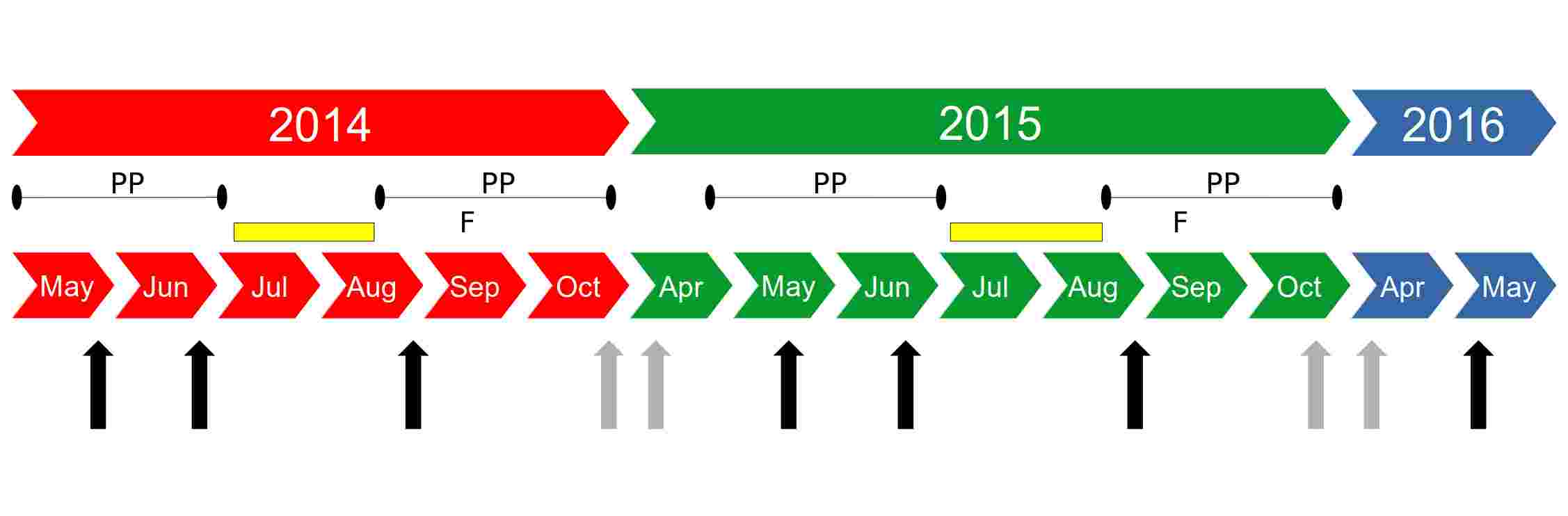

Supplement: toaf094_suppl_Supplementary_Materials [file toaf094_suppl_supplementary_materials.zip › Fig S1.tif]
