# Supplementary material for: Supersedure, mites, and visible disease in Apis mellifera (Hymenoptera: Apidae) colonies explain differences in productivity and survival, but the effects may be difficult to see
Source: J Econ Entomol. 2025 Jun 13;118(4):1463–74. doi: 10.1093/jee/toaf094 (PMC12397970; doi:10.1093/jee/toaf094)
Supplement: Supplementary file 7 [file toaf094_suppl_supplementary_materials.zip › Supplemental File Captions.odt]

Supplemental Files

Supplemental  Fig. S1. Timing of treatment applications and population assessments throughout the trial. PP indicates periods during which supplemental protein patties were applied. F indicates treatment with Fumagillin-B. Black arrows indicate full assessment dates. Grey arrows indicate simplified assessment dates before and after winter. Yellow bars indicate the honey production season (Alberta colonies only; colonies in Prince Edward Island were not used for honey production and received protein patties continuously).

Supplemental file 1. Statistical analysis output. This file provides the detailed the statistical analysis of the dataset, including full descriptions of the models and additional plots and tables.

Supplemental file 2. Statistical analysis code. This is the R markdown file with the exact statistical analysis code used to generate Supplemental file 1. Readable by R studio, provided a valid path to the project dataset is given at line 30.

Supplemental file 3. Project dataset.
